# Atelocollagen supports three-dimensional culture of human induced pluripotent stem cells

**DOI:** 10.1016/j.omtm.2024.101302

**Published:** 2024-07-20

**Authors:** Yoshiki Nakashima, Masayoshi Tsukahara

**Affiliations:** 1 CiRA Foundation, Research and Development Center, Nakanoshima Qross, Osaka 530-005, Japan

**Keywords:** human induced pluripotent stem cells, hiPSCs, three-dimensional culture, cell therapy using autologous iPSCs, atelocollagen beads, automated culture processes

## Abstract

As autologous induced pluripotent stem cell (iPSC) therapy requires a custom-made small-lot cell production line, and the cell production method differs significantly from the existing processes for producing allogeneic iPSC stocks for clinical use. Specifically, mass culture to produce stock is no longer necessary; instead, a series of operations from iPSC production to induction of differentiation of therapeutic cells must be performed continuously. A three-dimensional (3D) culture method using small, closed-cell manufacturing devices is suitable for autologous iPSC therapy. The use of such devices avoids the need to handle many patient-derived specimens in a single clean room; handling of cell cultures in an open system in a cell processing facility increases the risk of infection. In this study, atelocollagen beads were evaluated as a 3D biomaterial to assist 3D culture in the establishment, expansion culture, and induction of differentiation of iPSCs. It was found that iPSCs can be handled in a closed-cell device with the same ease as use of a two-dimensional (2D) culture when laminin-511 is added to the medium. In conclusion, atelocollagen beads enable 3D culture of iPSCs, and the quality of the obtained cells is at the same level as those derived from 2D culture.

## Graphical abstract

**Figure:**
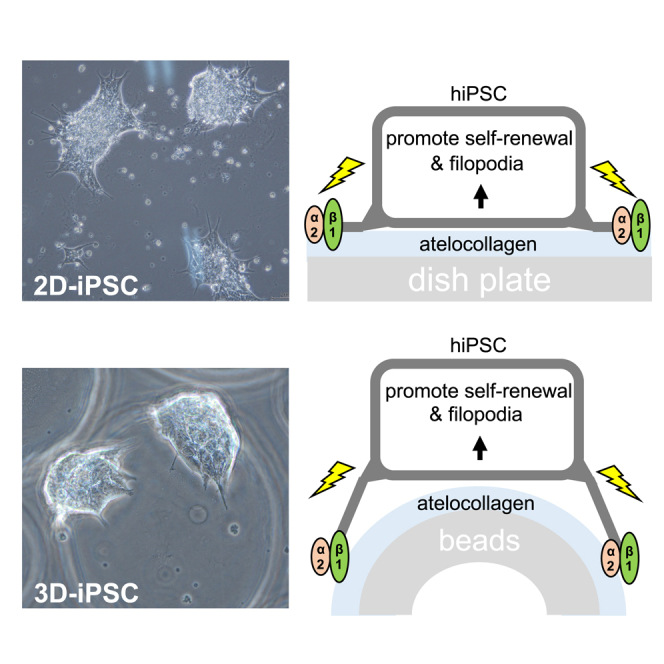

## Teaser

Nakashima and colleagues show that autologous iPSC therapy requires use of custom-made small-lot cell production lines. The 3D culture method using a small-type closed-cell manufacturing device is suitable for autologous iPSC therapy. Atelocollagen beads act as 3D biomaterials that assist in the establishment of iPSCs, expansion culture, and the differentiation induction process.

## Introduction

Currently, the standard approach for production of human induced pluripotent stem cells (hiPSCs) for clinical purposes is through use of two-dimensional (2D) cultures, i.e., cultures in which the cells are attached to a substrate coated with scaffold material.^1^^,^^2^ Although this approach provides viable hiPSCs, the method does have some limitations; it does not lend itself to upscaling for mechanized cell production using culture dishes or for automated culture processes for industrialized mass production of clinical iPSCs.^3^^,^^4^^,^^5^^,^^6^^,^^7^

Alternative 3D methods are available that use cell suspension in growth medium,^8^^,^^9^ cell growth on microcarriers,^10^ and cell suspension in polymer gels.^11^^,^^12^ The embryoid body (EB) method, which is based on the formation of pseudo-embryos in floating culture, is widely used for differentiation of cardiomyocytes,^13^^,^^14^ hepatocytes,^15^ neurons,^16^ and blood system cells, such as platelets^17^ and T cells^18^ from cultured iPSCs. Although the EB method can be used to culture differentiated cells, there can be problems in culturing undifferentiated iPSCs. EBs are not pseudo-embryos. Also, EBs are viewed as an *in vitro* model of gastrulation and are typically not used for production of high-quality iPSCs. Indeed, the production of high-quality, undifferentiated iPSCs for clinical use is difficult using the EB system,^19^ as the quality of cells is affected by the death of cells in the center of the EB and the induction of cell differentiation as the size of the EB increases.^12^^,^^20^

We have screened a number of candidate materials for 3D culture of hiPSCs. One of the most promising of these is atelocollagen, a low-immunogenic derivative of type I collagen obtained by removing the N- and C-terminal telopeptide components.^21^ Atelocollagen has an atomic mass of 300 kDa and has a rod-like structure with a length of 300 nm and a diameter of 1.5 nm. It has been clinically applied in various settings, such as wound healing, as a bone cartilage substitute and as a hemostatic agent.^22^ In the present report, we describe the characteristics and quality of hiPSCs cultured on atelocollagen. We also investigated the mechanism underlying their adhesion ability. To decrease the risk of rejection during cell therapy transplants, the ultimate goal is to generate iPSCs from the patient’s own cells, as this will enable the production of self-compatible differentiated cells that can be transplanted into the patient. Here, we sought to establish a cell manufacturing process using autologous iPSCs and focused on 3D cultures, as these have the desirable characteristic of supporting automated culture. This information has enabled us to propose a potential cell manufacturing process using atelocollagen.

## Results

### Manufacturing requirements for cell therapies using autologous iPSCs

**Figure 1:**
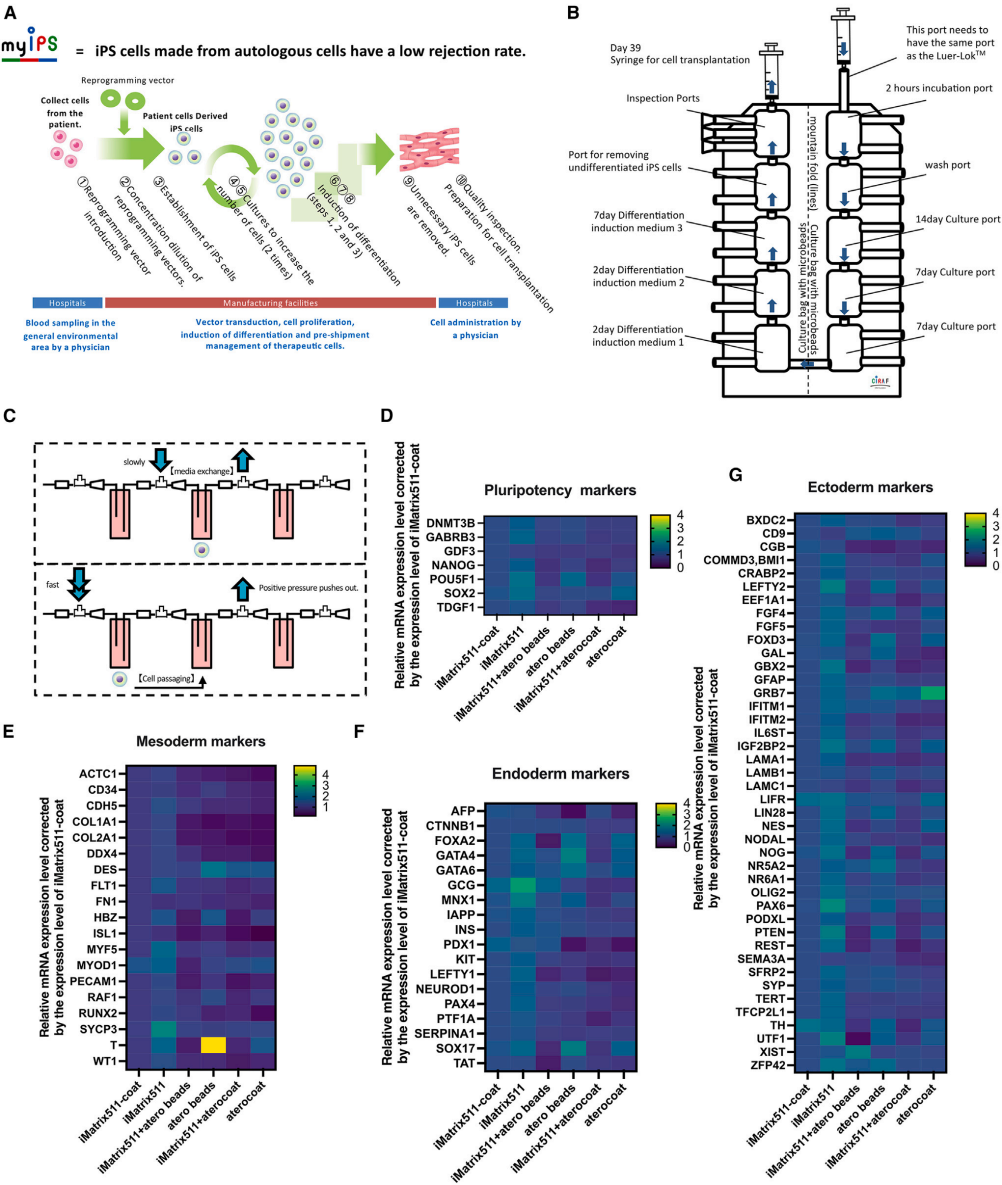
Concept and material requirements for cell therapy using autologous iPSCs Illustration of the work processes in cell therapy using autologous iPSCs (A). Illustration of a closed culture bag with small culture bags for each work process in cell therapy using autologous iPSCs (B). Illustration of medium exchange in a closed culture bag and cells moving between closed culture bags (C). mRNA expression analysis results from PBMC-derived iPSCs at day 6 after cell seeding at 1.3 × 10^4^ cells/well. Cultured iPSCs were seeded (1) on iMatrix-511-coated plates, (2) in iMatrix-511-containing medium, (3) in iMatrix-511-containing medium with atelocollagen beads, (4) with atelocollagen beads, (5) in iMatrix-511-containing medium on atelocollagen-coated plates, and (6) on atelocollagen-coated plates. The cDNAs were synthesized from iPSCs sampled 6 days after seeding. Expression levels were calculated using the ΔΔCt method. The expression of the target gene was normalized against the expression of the housekeeping gene. Data were normalized by converting the average expression of various mRNAs of iPSCs cultured on iMatrix-511-coat to 1. A real-time qPCR analysis of the pluripotency marker (D), expression of mesoderm marker (E), endoderm markers (F) and ectoderm marker (G) is shown (*n* = 6 independent and separate experiments).

Cell therapies using autologous iPSCs have advantages such as suppression of graft-versus-host disease induction and avoidance of immune rejection. In cases where the iPSCs are derived from other members of the other family—is an allogeneic transplant—then it is feasible to establish iPSCs from patient-derived cells that are human leukocyte antigen (HLA) homologous; genome editing can be used to remove HLA. As a result, HLA-matched master cell banks have been created and stockpiled by iPSC suppliers. However, cell therapy using autologous iPSCs does not require HLA matching but results in the creation of iPSCs that are tailor-made for each individual patient. Furthermore, the process of inducing the differentiation of iPSCs to produce therapeutic cells must be carried out continuously. Normally, cells are collected from patients in a medical facility, such as a hospital, and then transported to a cell manufacturing facility where the iPSCs are produced and differentiation is induced. The derived therapeutic cells are then administered to the patient in a medical facility (Figure 1A). We are preparing to launch a project for autologous iPSC-derived cell therapy under the official name of my iPS®, which collectively refers to all the various steps of this type of therapy. The production of iPSC master cell banks is usually based on mass culture systems in which 5–6 workers produce approximately 300 frozen vials at a time in a clean room under good manufacturing practice (GMP) conditions. By contrast, autologous iPSCs are produced as a single lot; thus, the establishment and differentiation of iPSCs are performed in small cultures. The use of closed culture vessels is desirable for this process as it avoids the risk of contamination between patients. We have devised a connected culture bag (Figure 1B) and culture vessels to enable the establishment of iPSCs from patient-derived cells, expansion culture, and differentiation induction to produce therapeutic cells in a series of closed vessels. When connected culture bags or culture vessels are used, cells need to be moved in medium when transferring cells to the next bag or vessel. In this case, the use of microcarriers, which have a slightly heavier specific gravity than the culture medium, makes it possible to change the culture medium if the fluid flows at a low speed (Figure 1C, top); if the fluid flows at high speed, the cells and microcarriers become suspended in the medium, making it possible to move the cells (Figure 1C, bottom). The most common material for microcarriers (culture beads) is hard plastic. However, for cell therapy using autologous iPSC lines, we propose the use of atelocollagen beads, which can be dissolved by collagenase.

We examined the effects of atelocollagen and of beaded atelocollagen on undifferentiated iPSCs using an mRNA expression analysis. Cultured iPSCs were seeded (1) on iMatrix-511-coated plates, (2) in iMatrix-511-containing medium, (3) in iMatrix-511-containing medium with atelocollagen beads, (4) with atelocollagen beads, (5) in iMatrix-511-containing medium on atelocollagen-coated plates, and (6) on atelocollagen-coated plates, sampled cells 6 days after seeding. The mRNA levels of various pluripotency markers (Figure 1D, each value is shown in Table S4), mesoderm markers (Figure 1E, each value is shown in Table S4), endoderm markers (Figure 1F, each value is shown in Table S4), and ectoderm markers (Figure 1G, each value is shown in Table S4) were measured by real-time PCR. If the mRNA level was 4-fold (or more) higher or lower, this was regarded as abnormal and plotted in yellow on the heatmap. The results showed that neither atelocollagen nor beaded atelocollagen affected the undifferentiated state of iPSCs. In addition, they did not affect the endodermal and ectodermal differentiation abilities of iPSCs. Atelocollagen did not affect the endoderm differentiation ability of iPSCs. The only difference identified was that the expression level of *T*, a mesoderm marker, was increased by more than 4-fold in the culture with beads alone compared with the control. However, by using iMatrix-511-containing medium, the expression level of *T* could be decreased. These results indicate that cultures using atelocollagen beads in a flow state in a closed culture vessel do not interfere with the maintenance of undifferentiated iPSCs. Furthermore, the expression levels of differentiation markers of endoderm, mesoderm, and ectoderm were not altered by the addition of iMatrix-511 to the culture medium.

When iMatrix-511 was added to the medium, iPSCs formed cell clumps when low-adhesion plates were used (Figure S1D). The mRNA expression level of undifferentiated markers in iPSCs decreased when cell clumps formed (Figure S1E). Therefore, it is possible to maintain the mRNA expression level of undifferentiated markers by cell adhesion of iPSCs on atelocollagen beads.

The scaffold component iMatrix-511, which is composed of α5, β1, and γ1 chains, binds to the integrins α3β1, α6β1, and α6β4 integrins. Vitronectin supports the maintenance of hPSCs through the αVβ5 integrin. An integrin αVβ1 has been described as a receptor for vitronectin.^23^ The results of the experiment are shown when vitronectin was added to the medium instead of iMatrix-511 (Figures S1F–S1I, each value is shown in Table S5). Compared with the vitronectin-coated medium, the addition of vitronectin to the medium resulted in clumped iPSC colonies as observed by optical microscope. The endoderm markers PAX4 and SOX17 and the ectoderm markers ZFP42 and PTEN tended to increase when vitronectin was added to the medium. The expression levels of several pluripotency markers, differentiation markers of endoderm, mesoderm, and ectoderm differentiation markers were not altered by the addition of vitronectin to the culture medium.

In the following sections, we describe in detail the effects of culturing iPSCs using atelocollagen and atelocollagen beads.

### hiPSC adhesion to atelocollagen by elongation of filopodia

**Figure 2:**
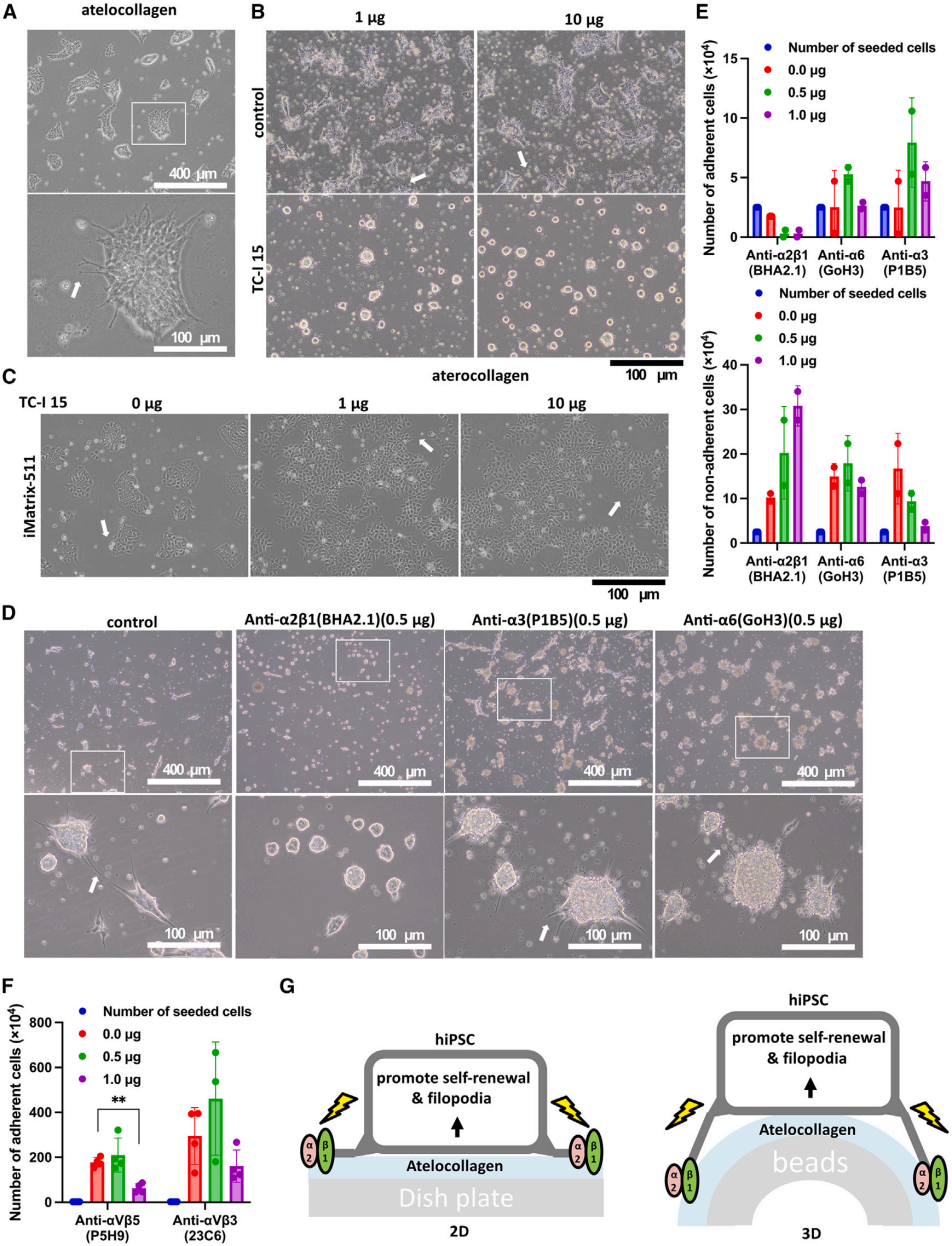
Elucidating the mechanism by which hiPSCs induce filopodia elongation as a reaction to atelocollagen Optical micrograph of hiPSCs attached to atelocollagen during filamentous elongation. White arrows indicate representative sites of filopodia. Scale bar, 400 μm (top) or 100 μm (bottom) (A). Optical microscope images 3 days after seeding of 15M66 cells at 2.5 × 10^4^ cells/well onto atelocollagen-coated wells with or without the reagent TC-I 15 at 1 or 10 μg/well. Scale bar, 100 μm. White arrows indicate representative sites of filopodia (B). Optical microscope images of 15M66 cells 3 days after seeding of 2.5 × 10^4^ cells/well onto iMatrix-511-coated wells with or without the reagent TC-I 15 at 1 or 10 μg/well. Scale bar, 100 μm. White arrows indicate representative sites of filopodia (C). Optical microscope images 3 days after seeding 15M66 cells at 2.5 × 10^4^ cells/well onto atelocollagen-coated wells with or without the reagents anti-α2β1 (BHA2.1), anti-α3 (P1B5), or anti-α6 (GoH3) at 0.5 μg/well. Scale bar, 400 μm (top) or 100 μm (bottom). White arrows indicate representative sites of filopodia (D). Live cell count data 3 days after seeding 15M66 cells at 2.5 × 10^4^ cells/well onto atelocollagen-coated wells with the reagents anti-α2β1 (BHA2.1), anti-α3 (P1B5), or anti-α6 (GoH3) at 0, 0.5, or 1.0 μg/well. The top shows the number of adherent cells. The bottom shows the number of non-adherent cells (*n* = 2 independent and separate experiments) (E). Live cells count data 9 days after seeding PBMC-derived iPSCs at 2.5 × 10^4^ cells/well onto atelocollagen-coated wells with the reagents anti-αVβ5 (P5H9) or anti-αVβ3 (23C6) at 0, 0.5, or 1.0 μg/well. Shows the number of adherent cells. ∗∗*p* < 0.01 (*n* = 4 independent and separate experiments) (F). Illustration of how atelocollagen induces the elongation of hiPSC filopodia. Atelocollagen activates integrin α2β1 in hiPSCs to activate self-renewal and filopodia. This mechanism works under 2D culture conditions (G, left) as well as under 3D culture conditions (G, right).

Optical microscope analysis of hiPSCs on atelocollagen showed the extension of extremely long filopodia (Figure 2A). We examined integrin α2β1, a receptor for collagen I, which is the main component of atelocollagen. TC-I 15, an integrin α2β1 inhibitor, was prepared as an additive reagent at 1 μg (dissolved in 0.1 μL DMSO). DMSO (0.1 or 1 μL) was used as a control. We seeded 15M66 line cells (2.5 × 10^4^ cells/well) onto iMatrix-511-coated plates (Figure 2B) and atelocollagen-coated plates (Figure 2C). In cultures that received 1 μg or 10 μg TC-I 15, cells on atelocollagen contained cell masses on day 3 of culture (Figure 2B). In control wells, some cell death occurred, but filopodia formation was observed (Figure 2B). This result indicates that the induction of filopodia formation by hiPSCs in atelocollagen is activated only by signals from integrin α2β1. In contrast, cells on iMatrix-511 in wells with 0, 1, or 10 μg TC-I 15 adhered and formed short filopodia on day 3 (Figure 2C). This result indicates that the induction of filopodia formation by hiPSCs on iMatrix-511 is not activated solely by signals from integrin α2β1.

TC-I 15 is not only specific to α2β1 but also shows inhibitory effects on αvβ3, α5β1, α6β1, and αIIbβ3. Therefore, it is difficult to conclude that α2β1 is the major factor involved in the cell adhesion of iPSCs to atelocollagen based on this experiment. We, therefore, investigated the effect of antibody-mediated inhibition of adhesion of iPSCs to atelocollagen. We analyzed cells at 3 days after seeding 15M66 line cells (2.5 × 10^4^ cells/well) onto atelocollagen-coated wells with or without antibodies against α2β1 (BHA2.1), α3 (P1B5), and α6 (GoH3) at 0.5 μg/well (Figure 2D). iPSCs cultured on atelocollagen showed more filamentous pseudopodia during culture with anti-α3 (P1B5) and anti-α6 (GoH3). In contrast, with anti-α2β1 (BHA2.1), iPSCs cultured on atelocollagen showed almost no filamentous pseudopodia elongation.

As iPSCs adhere to atelocollagen using filamentous pseudopodia, the number of adherent and non-adherent cells was measured to evaluate the degree of formation of filamentous pseudopodia. Live cell count data were obtained 3 days after seeding of 15M66 cells (2.5 × 10^4^ cells/well) onto atelocollagen-coated wells with various antibodies. The numbers of adherent cells are given in Figure 2E (top). Cultures containing the anti-α2β1 (BHA2.1) antibody had almost no adherent cells (Figure 2E, bottom). In contrast, the number of non-adherent cells increased by more than 8–12 times the number of seeded cells in cultures with anti-α2β1 (BHA2.1) antibody (Figure 2E, bottom).

Notably, the number of adherent cells increased by more than the number of seeded cells in cultures containing anti-α3 (P1B5) or anti-α6 (GoH3) antibodies (Figure 2E, top). In cultures with anti-α3 (P1B5) or anti-α6 (GoH3) antibodies, the numbers of non-adherent cells decreased compared with control cultures (Figure 2E, bottom). This indicates that the non-adhesion of iPSCs to atelocollagen did not occur in cultures with anti-α3 (P1B5) and anti-α6 (GoH3) antibodies. Integrins α3 and α6 are involved in the adhesion of iPSCs to laminin 511; the process by which iPSCs extend filopodia to achieve cell adhesion to atelocollagen is unlikely to involve laminin 511, which is an autocrine action by the iPSCs.

Live cells count data were obtained 9 days after seeding of peripheral blood mononuclear cell (PBMC)-derived iPSCs (2.5 × 10^4^ cells/well) onto atelocollagen-coated wells with anti-αVβ5 (P5H9) and anti-αVβ3 (23C6) antibodies. The number of adherent cells is shown in Figure 2F. The addition of 1.0 μg/well of integrin αVβ5 (P5H9) significantly decreased the number of iPSCs adhering to atelocollagen. These results suggest that vitronectin may support the maintenance of hPSCs on atelocollagen, as vitronectin supports hPSC maintenance via integrin αVβ5 integrin (Figure 2F).

The effect of atelocollagen on hiPSCs is illustrated in Figure 2: integrin α2β1 extends its filopodia and anchors hiPSCs to atelocollagen, not only under 2D conditions (Figure 2G, left) but also under 3D conditions (Figure 2G, right). The self-renewal of hiPSCs attached to atelocollagen is then promoted.

### Establishing hiPSCs on atelocollagen beads

The stealth RNA vectors (SRVs) iPSC-1, iPSC-2, iPSC-3, and iPSC-4 and CytoTune 2.0 can be used to establish of clinical iPSC cultures. However, SRVs were developed for cells grown on cell culture plates and are optimized for the establishment of hiPSCs under 2D conditions. We, therefore, investigated whether they could function in a similar manner in cultures using atelocollagen beads, a 3D environment.

**Figure 3:**
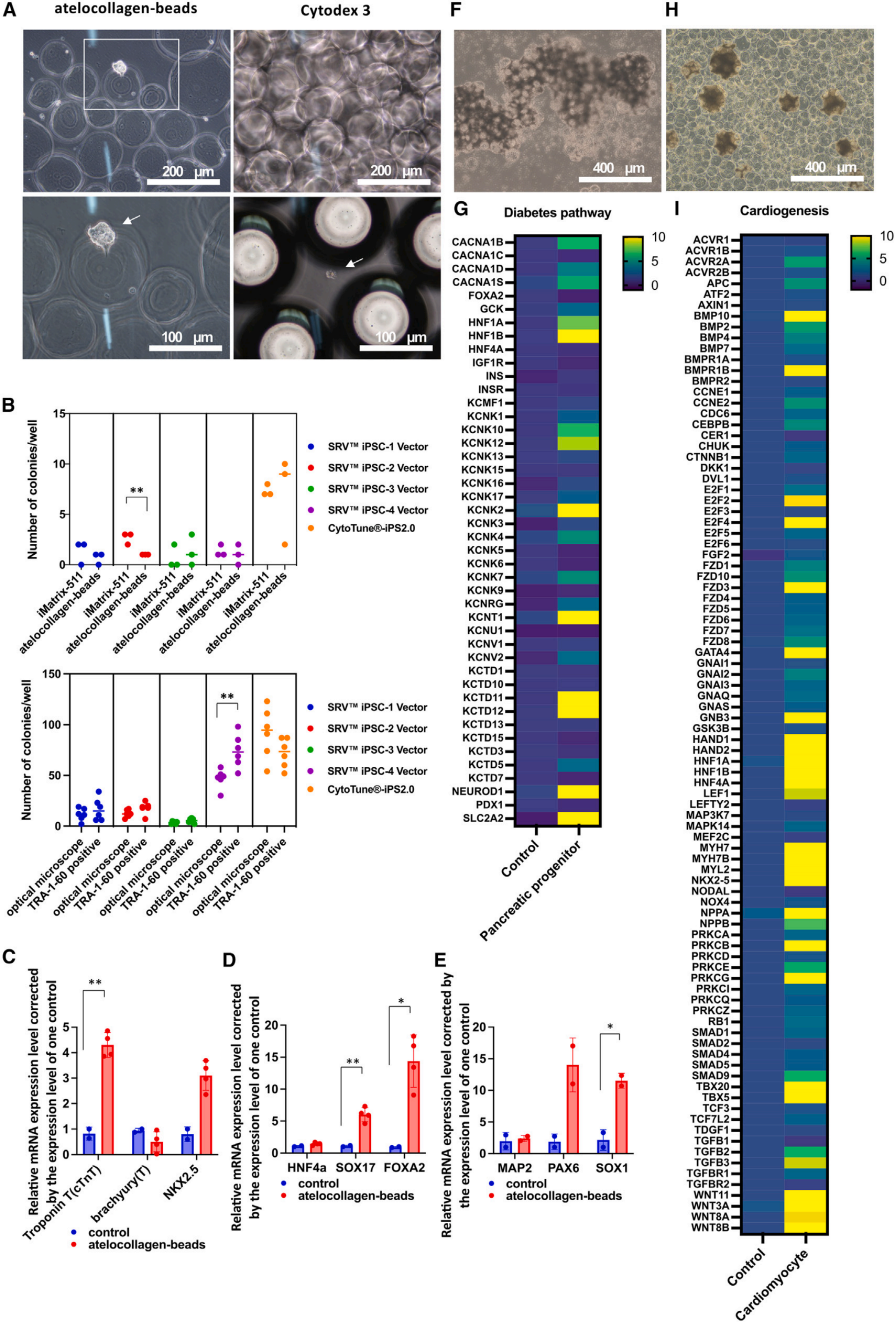
iPSCs were established on atelocollagen beads A total of 1 × 10^5^ human mononuclear cells were reprogrammed with the Sendai virus vector on atelocollagen beads or Cytodex3. Optical microscope image at day 15 after reprogramming. White arrows indicate established hiPSCs (A, left) or cell clumps that were incompletely established (A, right). A total of 1 × 10^5^ human mononuclear cells were reprogrammed with five different Sendai virus vectors (SRV iPSC-1, iPSC-2, iPSC-3, iPSC-4, and CytoTune 2.0) on iMatrix-511 or atelocollagen beads. Number of colonies/well at 15 days after reprogramming. ∗∗*p* < 0.01 (B, top). Re-experiments were conducted using atelocollagen beads as the scaffold material, and in addition to visual colony counts by optical microscopy, colony counts were also carried out by fluorescence microscopy with TRA-1-60 positivity as an indicator. Number of colonies counted by optical microscopy or counted as TRA-1-60 positive by fluorescence microscopy. ∗∗*p* < 0.01 (B, bottom). As a control group, cDNA was synthesized from eight passages of iPSCs established on atelocollagen beads. In the experimental group, cDNA was synthesized from hepatoblastoma cells, neural progenitor cells, and cardiac progenitor cells derived from the eighth passage of iPSCs established on atelocollagen beads. An expression analysis of each differentiation marker in mRNA sampled 11 days after the induction of differentiation of hiPSCs established on atelocollagen beads into cardiomyocytes (C), definitive endoderm (D), and neuroprogenitor cells (E) after eight passages on atelocollagen beads. Undifferentiated iPSCs from the eighth passage established on atelocollagen beads were used as a control to evaluate the differentiation induction ability. Expression was calculated using the ΔΔCt method. The expression of the target gene was normalized against expression of the housekeeping gene. The data were normalized by converting the average mRNA expression of various mRNAs in the eighth passage of iPSCs established on atelocollagen beads to 1. Data are shown as the mean ± SD (*n* = 4 independent and separate experiments). ∗*p* < 0.05. ∗∗*p* < 0.01. As a control group, cDNA was synthesized from cells after eight passages of iPSCs established on atelocollagen beads. In the experimental group, cDNA was synthesized from pancreatic progenitor cells and cardiomyocytes derived from the eighth passage of iPSCs established on atelocollagen beads. An expression analysis of each differentiation marker in mRNA sampled 22 days after the induction of differentiation of hiPSCs established on atelocollagen beads into pancreatic progenitor cells (G) and cardiomyocytes (I) after eight passages on atelocollagen beads. Photographs of pancreatic progenitor cells (F) and cardiomyocytes (H) 22 days after the start of differentiation induction are shown. Scale bar, 400 μm. Undifferentiated iPSCs from the eighth passage established on atelocollagen beads were used as a control to evaluate the differentiation induction ability. Expression was calculated using the ΔΔCt method. The expression of the target gene was normalized against expression of the housekeeping gene. The data were normalized by converting the average mRNA expression of various mRNAs of cells on day 0 of differentiation induction (undifferentiated) to 1 (*n* = 6 independent and separate experiments). Expression of diabetes pathway markers (G) and cardiogenesis markers (I).

Mononuclear cells (1 × 10^5^ cells) isolated from human blood were infected with a vector (SRV iPSC-2) at a multiplicity of infection (MOI) of 3 and the cells were seeded into six wells containing either atelocollagen beads (500 μL/well) or Cytodex 3 (500 μL/well). On day 15 of culture, hiPSC colonies were visually confirmed using an optical microscope. The hiPSCs had attached to the atelocollagen beads via filopodia (Figure 3A). In contrast, no colonies formed on the surface of the Cytodex 3 beads, and only cell clumps free from the microcarriers were present (Figure 3A). These findings indicated the possibility of establishing hiPSCs on atelocollagen beads.

Next, we compared the efficiency of hiPSC establishment on iMatrix-511 with that on atelocollagen using five different vectors for hiPSC establishment: SRV iPSC-1, iPSC-2, iPSC-3, and iPSC-4, and CytoTune 2.0. The efficiency of hiPSC establishment was assessed by counting the number of colonies in the wells using optical and fluorescence microscopy on day 14 after Sendai virus infection. We found no marked difference in the efficiency of hiPSC establishment between iMatrix-511-coated plates and atelocollagen for four of the five vectors; the exception was SRV iPSC-2 (Figure 3B, top). Next, iPSCs were established using atelocollagen beads as scaffold material. To confirm that the colonies identified by optical microscopy were iPSCs, the number of TRA-1-60-positive colonies was counted using fluorescence microscopy. For the SRV iPSC-4 vector, the number of TRA-1-60-positive colonies confirmed by fluorescence microscopy was significantly higher than the number of colonies confirmed by optical microscopy. For the other vectors used, the number of colonies identified by optical microscopy was comparable with that identified as TRA-1-60 positive by fluorescence microscopy (Figure 3B, bottom). Thus, atelocollagen beads are capable of acting as a scaffolding material for the establishment of iPSCs in 3D conditions.

KaryoStat was performed, and fifth-generation iPSCs established from both PBMCs on atelocollagen beads using SRV iPSC-2 showed no abnormalities in whole-genome KaryoStat (Figures S1A and S1B). Fifth-generation iPSCs established on atelocollagen beads using SRV iPSC-2 vector showed no abnormalities in whole-genome KaryoStat. A value of 2 represents an abnormal copy number (CN 2) state, a value of 3 represents chromosomal gain (CN 3), and a value of 1 represents a chromosomal loss (CN 1).

A PluriTest was performed,^24^^,^^25^ and all four passages of iPSCs established on atelocollagen from PBMCs using SRV iPSC-2 passed with gene expression values similar to the pluripotent stem cell group on the plot (Figure S1C). iPSCs established on atelocollagen show pluripotency.

We concluded that there was no marked difference in the efficiency of establishing hiPSCs on iMatrix-511-coated plates to that of hiPSCs on atelocollagen. The hiPSCs established on atelocollagen beads were trypsin-treated to detach the cells, which were then transferred to plates containing new atelocollagen beads (100–400 μm, approximately 3 million particles/15 mL) (500 μL/well) and then cultured further. To evaluate the pluripotency of the cells after eight cell passages, mRNA was extracted from cardiomyocytes (Figure 3C), definitive endoderm (Figure 3D), and neuroprogenitor cells (Figure 3E) at 11 days after differentiation induction, and their abilities to differentiate into three germ layers were evaluated. We used iPSCs from day 0 of induction of differentiation as controls. Quantitative comparisons of the mRNA data were performed using the ΔΔCT method, and the average value of the control was converted to 1. The maximum cycle threshold (CT) value was set at 40, and the calculation was performed by substituting 40 for the maximum CT value for undetected targets. First, we assessed pancreatic progenitor cells (Figures 3F and 3G) and cardiomyocytes (Figures 3H and 3I) after eight cell passages. We extracted mRNAs from pancreatic progenitor cells (Figure 3G) and cardiomyocytes (Figure 3I) at 22 days after the induction of differentiation. Differentiation and maturation of pancreatic progenitor cells was investigated by analyzing expression of factors involved in the maturity onset diabetes of the young *Homo sapiens* (human) pathway (https://www.genome.jp/pathway/hsa04950) (Kyoto Encyclopedia of Genes and Genomes). Key factors in pancreatic progenitor cell differentiation maturation were *HNF1α*, *HNF4α*, *HNF1β*, *GCK* (*GK*), *NEUROD1*, and *PDX1*. The levels of *HNF1A*, *HNF-1β*, *GCK*, and *NEUROD1* mRNAs were significantly increased in pancreatic progenitor cells induced to differentiate after eight passages of iPSCs in comparison with undifferentiated iPSCs after eight passages (Figure 3G; Table S1). An optical microscope analysis showed that pancreatic progenitor cells induced to differentiate after eight passages of iPSCs migrated and adhered to atelocollagen beads (Figure 3F).

The heart is formed through numerous developmental steps, including the determination of the cardiac field in the mesoderm, the differentiation of cardiac precursor cells, and cell maturation in the heart. The levels of *Tbx5*, *Tbx20*, *HAND1*, *HAND2*, *BMP2*, *BMP7*, *BMP10*, *WNT3A*, *WNT8A*, *WNT8B*, and *WNT11* mRNAs were significantly increased in cardiomyocytes induced to differentiate after eight passages of iPSCs in comparison with undifferentiated iPSCs after eight passages (Figure 3I; Table S2). An optical microscope analysis showed that cardiomyocytes induced to differentiate after eight passages of iPSCs migrated and adhered to atelocollagen beads (Figure 3H).

### Atelocollagen can be used in automated culture equipment

Clinical cell culture systems produce cells for clinical use under GMP standards and control. They culture cells under aseptic conditions and allow gene manipulation, initialization, proliferation, differentiation induction, and freezing, among other activities.

**Figure 4:**
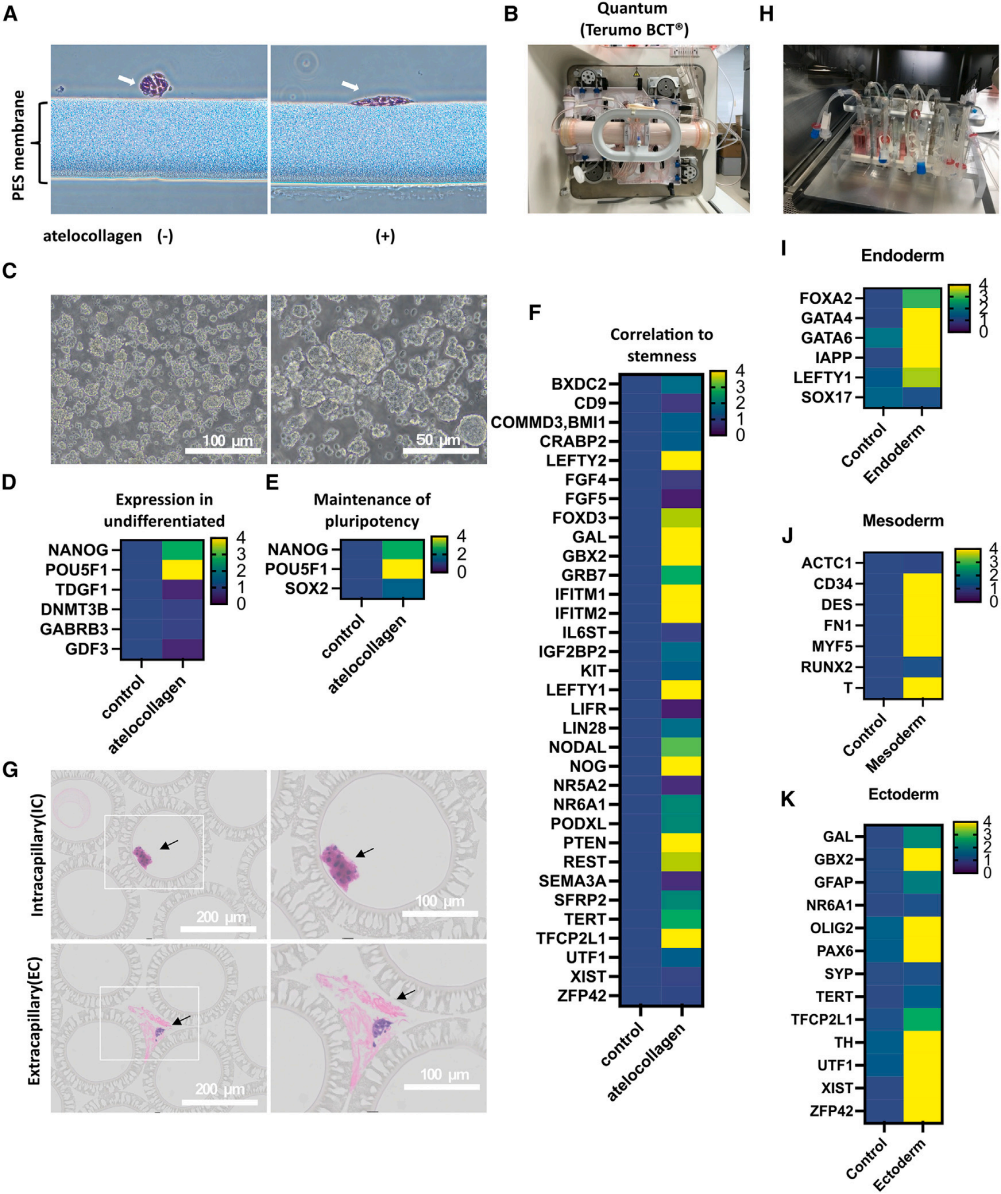
hiPSCs cultured in bioreactors with hollow fiber membranes of PES material coated with atelocollagen Optical microscope images (×400) of 15M66 cells (white arrows) adhered to PES membranes without atelocollagen coating (A, left) or with atelocollagen coating (A, right). Photograph of an automated cell culture device using a bioreactor with hollow fiber membrane membranes of PES material (B). Optical microscope image of cells collected after detachment with collagenase on day 4 after seeding of 15M66 cells at 5 × 10^5^ cells/well into a bioreactor with hollow fiber membranes of PES material (C). The results of an mRNA analysis of cells collected after detachment with collagenase on day 4 after seeding of 15M66 cells at 5 × 10^5^ cells/well into a bioreactor with hollow fiber membranes of PES material. cDNA were synthesized from iPSCs cultured on iMatrix-511 and iPSCs cultured onto bioreactors with hollow fiber membranes of PES material. Expression was calculated using the ΔΔCt method. Expression of the target gene was normalized against the expression of the housekeeping gene. Data were normalized by converting the average expression of various mRNAs of iPSCs cultured on iMatrix-511 to 1. Expression of undifferentiated cell markers (D), maintenance of pluripotency markers (E), and correlation with stemness markers (F) (*n* = 1 replicate plates in 1 experiment). Observation of hiPSCs cultured in a bioreactor with atelocollagen-coated hollow fiber membranes. HE staining was applied to IC and EC sections. Colonies of hiPSCs (black arrows). Scale bars indicate the designated lengths (G). Self-made closed system culture vessel with 20 mL of liquid content. Closed culture vessels can connect to the outside world via the attached tubing and the intake of outside air via an air filter (H). 15M66 cells were seeded at 1 × 10^6^ cells into closed culture vessels with 5 mL of atelocollagen bead suspension (MIC-00) and 10 mL of StemFit AK03 with 10 μM Y-27632. From day 3 after the start of culture, the medium in the closed culture vessel was replaced with new medium, and differentiation induction into endoderm (5 days), mesoderm (5 days), and ectoderm (7 days) was initiated. After differentiation induction had been completed, cells were detached with collagenase, and an mRNA analysis of the recovered cells was conducted. The mRNA expression of cells on day 0 of differentiation induction (undifferentiated) was used as a control (average of three samples was used). cDNA was synthesized from iPSCs (strain 15M66) induced to differentiate in a closed culture system. Expression was calculated using the ΔΔCt method. Expression of the target gene was normalized against the housekeeping gene expression. Data were normalized by converting the average expression of the various mRNAs of cells on day 0 of differentiation induction (undifferentiated) to 1. Expression of endoderm markers (I), mesoderm markers (J), and ectoderm markers (K).

Currently, the hollow fiber membrane material used in cell bioreactors is mainly polyethersulfone (PES); unfortunately, hiPSCs do not bind readily to this material. Atelocollagen is a liquid with good flow at a pH 3 of but hardens to a jelly-like state at a neutral pH. Therefore, it is possible to create a hybrid layer of atelocollagen on PES membranes by injecting atelocollagen (pH of 3) as a liquid onto the PES membrane and then changing to neutral conditions using PBS or culture medium. Although 15M66 line cells do not adhere to a PES membrane (Figure 4A, left), they will adhere to atelocollagen-coated PES membranes (Figure 4A, right). We seeded 15M66 line cells (5 × 10^5^ cells) into a bioreactor with an atelocollagen-coated hollow fiber membrane and cells were detached from the membrane 4 days later using collagenase (Figure 4C). Since the cells were detached with collagenase, cell-cell adhesion was maintained, and the cells were able to be sampled as a cell mass (cell viability, 80%). mRNA was extracted for an expression analysis. The following categories of mRNAs were assessed using the TaqMan Human Stem Cell Pluripotency Array (Applied Biosystems): expression in undifferentiated cells (Figure 4D), maintenance of the list categorized into pluripotency (Figure 4E), and correlation to stemness (Figure 4F) were analyzed. The mRNA expression was color coded using cells cultured on iMatrix-511-coated plates as controls. The results showed that cells cultured on the atelocollagen-coated PES hollow fiber membrane had higher expression of *OCT3/4* (*POU5F1*) (Figures 4D and 4E), indicating a trend toward an increased undifferentiated state. The 15M66 line cells cultured in an atelocollagen-coated PES hollow fiber membrane bioreactor showed increased expression of *LEFTY2* (Figure 4F), which is involved in left-right asymmetry of developing organ systems. On day 7 of culture, hollow fiber membranes in the bioreactor were removed and paraffin embedded, and tissue sections were prepared for microscopic examination. Hematoxylin and eosin (HE) staining was used to stain intracapillary (IC) and extracapillary (EC) sections of the hollow fiber membrane on day 7 of culture. Colonies of hiPSCs were identified (Figure 4G).

Next, to perform differentiation induction tests, we created a closed culture vessel that had a 20-mL capacity, and in which liquid could be delivered externally via a tube. In addition, external air could be transferred via an air filter (Figure 4H). Undifferentiated iPSCs were cultured on atelocollagen beads in the 20-mL closed vessel, and then 10 mL of StemFit AK03 (Ajinomoto) containing 10 μM Y-27632 (Fujifilm Wako), 5 mL atelocollagen bead solution (MIC-00; KOKEN CO., LTD), and 1 × 10^6^ 15M66 cells for 3 days. On day 3 of culture, endoderm differentiation induction was initiated by adding 20 mL STEMdiff Trilineage Endoderm Medium. Cells were sampled on day 5 of differentiation induction. The mRNA expression of cells on day 0 of differentiation induction (undifferentiated) was used as a control (average of three samples was used). The endoderm marker forkhead box protein A2 (*FOXA2*) was increased 2.95-fold compared with controls. The endoderm marker GATA binding protein 4 (*GATA4*) was increased 62.73-fold compared with controls, and GATA binding protein 6 (*GATA6*), a marker of the endodermis, was increased 30.32-fold compared with the control (Figure 4I). To determine whether mesoderm induction could be achieved, we added 20 mL STEMdiff Trilineage Mesoderm Medium into a separate culture vessel. Cells were sampled on day 5 of differentiation induction. The mRNA expression of cells on day 0 of differentiation induction (undifferentiated) was used as a control (average of three samples was used). The mesoderm marker *T* (*Brachyury*) was increased 766.14-fold in cells on day 5 of differentiation induction compared with controls (Figure 4J). Finally, we added 20 mL STEMdiff Trilineage Ectoderm Medium on day 3 of culture to initiate ectoderm differentiation. Cells were sampled on day 7 after differentiation induction. The mRNA expression of cells on day 0 of differentiation induction (undifferentiated) was used as a control (average of three samples was used). The ectoderm marker paired box 6 (*PAX6*) was increased 18.24-fold in cells on day 7 of differentiation induction compared with controls (Figure 4K).

These results indicate that iPSCs cultured on atelocollagen beads in closed vessels can undergo 3D embryonic differentiation, showing markedly increased marker levels in each embryo. In conclusion, atelocollagen offers a viable scaffolding material for inducing iPSC differentiation under 3D culture conditions.

## Discussion

In this article, we described the use of atelocollagen as a material option for establishing hiPSCs for clinical use and inducing the differentiation of therapeutic cells in a 3D culture environment.

Three-dimensional culture methods for hiPSCs include 3D suspension in growth medium,^8^^,^^9^ growth on microcarriers,^10^ and suspension in polymer gels.^11^^,^^12^ The EB method, which is based on the formation of pseudo-embryos (EBs) in floating culture, is also widely used for cardiomyocyte differentiation,^13^^,^^14^ hepatocytes,^15^ neurons,^16^ and blood system cells, such as platelets^17^ and T cells.^18^ However, it is difficult to control the culture conditions of undifferentiated iPSCs for clinical use, which must be maintained at a high level of quality,^19^ as the quality of cells is affected by the death of cells in the center and accelerated differentiation induction as the size of the EB increases.^12^^,^^20^ Notably, the EB method is a complete floating culture method. An intermediate culture method between the EB method and the adhesion culture method is the semi-floating culture method of hiPSCs, which uses floating units, such as microcarriers.^26^ Researchers have adopted a technique involving the encapsulation of hiPSCs in a 3D container that allows them to be cultured in an environment as close to 2D as possible. Examples of such methods include tubing^27^ and encapsulation.^28^^,^^29^ Some technologies have been reported to successfully achieve a semi-floating method of hiPSC culture by imparting standing peptide chains,^30^ such as Synthemax II (Corning Incorporated).

There are two types of methods for culturing iPSCs under 3D conditions: culturing iPSCs by cell adhesion to the surface of a suspended structure and culturing iPSCs as a cell mass in a nonadherent manner. The first method uses the Synthemax II microcarrier. The microcarrier structure involves a Synthemax II polymer attached to a polystyrene microcarrier.^31^ The second method involves floating iPSC clusters in methylcellulose-containing medium^12^ or PNIPAAm-PEG-containing medium, which is a hydrogel.^32^

We have attempted 3D cultures with many materials over the past few years, believing that hiPSCs are indeed suitable for autonomous 3D culture. As a result, we discovered the existence of hiPSCs extending strangely long filopodia on atelocollagen. Atelocollagen is a low-immunogenic collagen derivative obtained by removing the N- and C-terminal telopeptide components, which are known to induce antigenicity in humans,^21^ by treating type I collagen with pepsin. The resulting atelocollagen (300 kDa) has a rod-like structure with a length of 300 nm and a diameter of 1.5 nm. The advantage of atelocollagen over other regenerative medicine materials is that it has already been clinically applied in various settings, such as wound healing, bone cartilage substitutes, and hemostatic agents. In recent years, it has also been reported to be highly versatile as a capsule material for drug delivery in gene therapy^21^^,^^33^^,^^34^ and other applications.

In this study, we investigated the use of atelocollagen as a culture material for establishing hiPSCs for clinical use and inducing the differentiation of therapeutic cells in a 3D culture environment. The iPSCs we describe were not established for use in allogeneic transplantation or to create a master cell bank. The study described here is part of our current attempts to use automated culture to produce clinical iPSCs that can be used in autologous therapy. It is known that, as the number of passages increases, there is a greater the risk of DNA mutations. Therefore, autologous iPSCs are cultured in relatively small quantities, obviating the need for a large number of passages. Instead, the induction of differentiation is initiated shortly after iPSCs are established. Consequently, we assume that iPSCs will be passaged no more than five times. Autologous iPSCs are expensive to produce manually, so automated cultivation is considered desirable. The use of 3D culture systems allows the cells to be produced in closed culture devices.

Since iPSCs are in a floating state under 3D culture conditions, they are more susceptible to physical stimuli than in adherent cultures. Therefore, they are more likely to differentiate; we found that culturing iPSCs on atelocollagen beads resulted in an increased level of expression of *T* mRNA in the mesoderm (Figure 1E). The addition of iMatrix-511 to the culture medium reduced the susceptibility to induction of differentiation (Figure 1E). Based on these results, we conclude that iMatrix-511 should be added to the culture medium for 3D culture of iPSCs. The quality of iPSCs produced in 3D culture with atelocollagen beads and iMatrix-511-supplemented medium was comparable to that of iPSCs cultured under 2D conditions on iMatrix-511-coated plates. This may because the effect of atelocollagen on iPSCs is limited to cell adhesion via integrin α2β1 (Figures 2B and 2D), which has a limited effect on cell quality.

## Materials and Methods

### Cell lines

#### Maintenance culture of human mononuclear cells

Normal human PBMCs-Japanese donor, purified-characterized (10 M cells/vial) were obtained from FUJIFILM Wako Pure Chemical Corporation. The medium for human mononuclear cells consisted of stem cell factor/c-Kit ligand (final concentration 50 ng/mL), thrombopoietin (final concentration 10 ng/mL), Flt3L (final concentration 20 ng/mL), interleukin (IL)-6 (final concentration 50 ng/mL), IL-3 (20 ng/mL), and granulocyte colony stimulating factor (10 ng/mL) in a mixture of liquids A and B of StemFit AK03N (Ajinomoto Healthy Supply Co., Inc.).

The following protocol is a method for culturing PBMCs and is a brief description of the procedure normally followed at our institution. Thaw a frozen vial of normal human PBMCs in a 37°C water bath for 1 min. Suspend the PBMCs in 5 mL of a mixture of liquids A and B of StemFit AK03N, and then centrifuge the sample (440 × *g* for 5 min at 22°C). After removing the supernatant, add 1 mL of culture medium for human mononuclear cells, mix, and count the cells. A total of 3 × 10^6^ cells/mL culture medium for human mononuclear cells was used in our case. Cells are seeded in 24-well plates at a volume of 1 mL/well and incubated at 37°C for 5 days at 20% O_2_ and 5% CO_2_.

#### Establishment of hiPSCs

hiPSCs were established using the TOKIWA-Bio SRV iPS-1 Vector, Tokiwa-Bio SRV iPSC-2, Tokiwa-Bio SRV iPS-3 Vector, and Tokiwa-Bio SRV iPSC-4 according to the manufacturer’s instructions (Tokiwa-Bio Inc.). In brief, 1 × 10^5^ cells were dispensed into microtubes and centrifuged (300 × *g* for 5 min). After removing the supernatant, 10 μL of the vector supplied in the kit was added. Another 10 μL of human mononuclear cell culture medium was then added, and the solution was incubated at 37°C for 2 h at 20% O_2_ and 5% CO_2_. Centrifugation was repeated (300 × *g* for 5 min), followed by three washes in human mononuclear cell culture medium. Culture with human mononuclear cell medium was then initiated; two-thirds of the volume of StemFit AK03N medium was added on days 1, 3, 5, and 7 of culture at 37°C for 2 h at 20% O_2_ and 5% CO_2_. The medium was replaced with StemFit AK03N medium on days 9, 11, and 13 after culture initiation. Cell passaging and colony picking were performed from day 15 of culture.

hiPSCs were established using CytoTune-iPS 2.0 Vector, according to the manufacturer’s instructions (ID Pharma Co., Ltd.). In brief, 1 × 10^5^ cells were dispensed into microtubes and centrifuged (300 × *g* for 5 min). In accordance with the data sheet attached to the kit, 7.14 μL of Tube KOS, 6.66 μL of Tube KLF4, and 10.00 μL of Tube C-MYC, the vector included with the kit, were added to 2 mL of medium for human mononuclear cells. A total of 1 × 10^5^ cells were then seeded onto a six-well plate. The cells were cultured in human mononuclear cell medium supplemented with various types of vectors (MOI = 5) at 37°C for 2 h at 20% O_2_ and 5% CO_2_. Next, two-thirds of the volume of StemFit AK03N medium was added on days 1, 3, 5, and 7 of culture at 37°C for 2 h at 20% O_2_ and 5% CO_2_. The medium was exchanged with StemFit AK03N medium on days 9, 11, and 13 of culture. Cell passaging and colony picking were performed from day 15 of culture.

#### Maintenance culture of hiPSCs

The hiPSC lines 15M66 were established by Shinya Yamanaka (CiRA Foundation) and obtained from CiRA Foundation. The iPSCs were cultured using a publicly available method (CiRA_Ff-iPSC_protocol_Eng_v140310) (https://www.cira.kyoto-u.ac.jp/j/research/img/protocol/Ff-iPSC-culture_protocol_E_v140311.pdf).^6^^,^^35^ Coating with iMatrix-511 (175 μg/0.35 mL/tube) was performed by adding 9.6 μL to each well of a six-well plate. The iMatrix-511 (9.6 μL) was first diluted in 1.5 mL PBS and then added as the coating solution to each well of a six-well plate. A coating time of at least 1 h at 37°C was used. iMatrix-511 (175 μg/0.35 mL/tube)-containing medium was prepared by diluting a total of 4.8 μL iMatrix-511 into 10 mL of StemFit AK03N medium.

#### Quantum cell growth system

The Quantum System (Terumo BCT) is an automated, functionally closed system integrating culture, gas supplies, and fluid handling for the management of hollow fiber bioreactors. Operation of the Quantum System involves several steps that must be performed in a safety cabinet. It is necessary to fill the bags with medium and reagents (e.g., medium, PBS, cells, coating solution, cell detachment solution) using a peristaltic pump (07528-10; Masterflex). These bags are connected to the Quantum System via a sterile connection device (TSCD-II, Terumo BCT), and the system is controlled via a touch screen interface. The system consists of a synthetic hollow fiber bioreactor that is part of a sterile closed loop circuit for medium and gas exchange. This bioreactor culture system is a one-time disposable set. Standard conditions for culture are maintained, including an incubation temperature of 37°C and a mixed gas supply (0.3 MPa) with 5% CO_2_ and 20% O_2_ supplied and balanced with N_2_.

The Quantum System was prepared according to the manufacturer’s protocol for inserting and priming disposable Cell Expansion Sets (including hollow fiber bioreactors) into the Quantum System.^36^^,^^37^^,^^38^

##### Culture surface area coating

Before loading cells, the culture surface area of the hollow fiber bioreactor must be coated. For this purpose, 100 mL Atelocollagen Acidic Solution (5 mg/mL, pH 3.0) (Koken Co., Ltd.) was loaded into the Quantum System. After the priming process, the atelocollagen solution was loaded into the IC at an inflow rate of 10 mL/min and coated at an IC circulation flow rate of 10 mL/min. Next, StemFit AK03N medium containing 10 μM Y-27632 was loaded into the IC at an inflow rate of 10 mL/min, and the atelocollagen was cured in the hollow fiber bioreactor at a flow rate of 10 mL/min for 30 min. If the IC flow rate was stopped within 30 min during the coating process for risk management, the flow path in the hollow fiber bioreactor became blocked (internal pressure exceeded 700 mm Hg, and an alarm sounded). However, even after the blockage, it was possible to manually switch the IC circulation flow on and off continuously to return to normal operation with an internal pressure of 250–300 mm Hg within approximately 1 h.

##### Cultivation of hiPSCs in the bioreactor

The Quantum System was seeded with 5 × 10^5^ hiPSCs suspended in 100 mL of StemFit AK03N medium containing 10 μM Y-27632 or CTS Essential 8 Medium (Thermo Fisher Scientific K.K.) containing 10 μM Y-27632. The flow rates used were 150 mL/min IC circulating flow rate and 30 mL/min EC circulating flow rate. Cells were allowed to adhere for 12 h at an IC circulating flow rate of 0.1 mL/min and an EC circulating flow rate of 0.1 mL/min. Perfusion was then automatically started at 0.2 mL/min, and the cells were cultured for 4 days. HE staining was performed at Biopathology Institute Co., Ltd.

##### Harvesting of hiPSCs from bioreactors

The harvesting process involves washing the system with PBS, adding 180 mL collagenase, 1 g/PBS 180 mL, incubating for 15 min, and then flushing the cells into harvest bags using StemFit AK03N medium containing Y-27632.

#### Differentiation assay to three germ layers

##### Cardiomyocyte differentiation

To induce differentiation into myocardial cells, hiPSCs were cultured to confluence in six-well plates in StemFit AK03N medium on a support using a PSC Cardiomyocyte Differentiation Kit, according to the manufacturer’s instructions (https://www.thermofisher.com/document-connect/document-connect.html?url=https://assets.thermofisher.com/TFS-Assets%2FLSG%2Fmanuals%2FMAN0014509_psc_cardiomyocyte_diff_PI.pdf) (Thermo Fisher Scientific K.K.) or STEMdiff Cardiomyocyte Differentiation and Maintenance Kits, according to the manufacturer’s instructions (https://cdn.stemcell.com/media/files/pis/DX21496-PIS_1_0_0.pdf?_ga=2.262974152.598384201.1535506696-776122060.1533191873) (Stem Cell Technologies Inc.). Specifically, after culturing for two days using the A solution included in the kit, further culture was performed for another 2 days using the B solution. The myocardial differentiation potential was then evaluated using cells that had been cultured in solution C for 5–7 days. Culture was performed in an incubator at 37°C with 20% O_2_ and 5% CO_2_.

##### Definitive endoderm differentiation

To induce differentiation into hepatoblasts, hiPSCs were cultured to confluence in six-well plates in StemFit AK03N medium. The induction of differentiation of definitive endoderm^39^ followed the previously reported protocol. In brief, endoderm differentiation was performed using media of the following compositions:

[Day 1]
  Medium: RPMI 1640 with GlutaMAX + B27 (-insulin) + NEAA (1%) with activin A (100 ng/mL), BMP4 (50 ng/mL) and CHIR99021 (3 μM). Culture was performed in an incubator at 37°C with 20% O_2_ and 5% CO_2_.
[Days 2–7]
  Medium: RPMI 1640 GlutaMAX + B27 (-insulin) + NEAA (1%) with activin A (100 ng/mL) and BMP4 (50 ng/mL). The medium was changed daily. Culture was performed in an incubator at 37°C with 20% O_2_ and 5% CO_2_.
[Days 8–10]
  Medium: RPMI 1640 with GlutaMAX + B27 (+insulin) + NEAA (1%) with basic fibroblast growth factor (10 ng/mL), BMP4 (50 ng/mL) and HGF (10 ng/mL). The medium was changed daily. Culture was performed in an incubator at 37°C with 20% O_2_ and 5% CO_2_.

##### Neuroprogenitor cell differentiation

To induce differentiation to neuroprogenitor cells, hiPSCs were cultured to confluence in six-well plates in StemFit AK03N medium on a support using a PSC Neural Induction Medium, according to the manufacturer’s instructions (https://www.thermofisher.com/document-connect/document-connect.html?url=https://assets.thermofisher.com/TFS-Assets%2FLSG%2Fmanuals%2Fpsc_neural_induction_medium_man.pdf) (Thermo Fisher Scientific K.K.) or STEMdiff SMADi Neural Induction Kit & STEMdiff Neural Progenitor Medium, according to the manufacturer’s instructions (https://cdn.stemcell.com/media/files/pis/10000000231-PIS_04.pdf; https://cdn.stemcell.com/media/files/pis/10000003488-PIS_01.pdf) (Stem Cell Technologies Inc.). Specifically, after culturing for 11 days in the PSC Neural Induction Medium included in the kit, neural differentiation potential was evaluated. After culturing for 8 days in the STEMdiff Neural Induction Medium included in the kit, and after culturing for 12 days in the STEMdiff Neural Progenitor Medium, the neural differentiation potential was evaluated. Culture was performed in an incubator at 37°C with 20% O_2_ and 5% CO_2_.

##### Pancreatic progenitor differentiation

To induce differentiation of pancreatic progenitor cells, hiPSCs were cultured to confluence in six-well plates in StemFit AK03N medium on a support using a STEMdiff Pancreatic Progenitor Kit, according to the manufacturer’s instructions (https://cdn.stemcell.com/media/files/pis/DX20464-PIS_1_3_0.pdf?_ga=2.9554801.598384201.1535506696-776122060.1533191873) (Stem Cell Technologies Inc.). After culturing for one day using the Medium 1A solution included in the kit, the cells were further cultured for 1 day using the Medium 1B solution, 1 day using the Medium 2A solution, 2 days using the Medium 2B solution, and 3 days using the Medium 3 solution. The pancreatic progenitor differentiation potential was then evaluated using cells that had been cultured in Medium 4 solution for 14 days. Culturing was performed in an incubator at 37 C with 20% O_2_ and 5% CO_2_.

##### Trigeminal differentiation

Closed-system culture vessels with 20 mL liquid volume were manufactured by Tokai Hit., Co, Ltd (Shizuoka, Japan). A STEMdiff Trilineage differentiation kit (ST-05230; (Stem cell Technologies), was used. Differentiation of iPSCs into ectoderm was induced by culturing cells for 7 days using STEMdiff Trilineage ectoderm medium. Differentiation of iPSCs into mesoderm was induced by culturing cells for 5 days using STEMdiff Trilineage Mesoderm Medium. Differentiation of iPSCs into endoderm was induced by culturing cells for 5 days using STEMdiff Trilineage Endoderm Medium.

#### Real-time PCR

RNA was prepared using a SuperPREP II Cell Lysis & RT Kit for quantitative PCR (Toyobo Co., LTD.) according to the manufacturer’s instructions. Real-time PCR was performed using a StepOnePlus system (Life Technologies). Luna Universal qPCR Master Mix (New England Biolabs Inc.) was used according to the manufacturer’s instructions. The PCR protocol was as follows: (1) initial denaturation at 95°C for 10 min; (2) denaturation at 95°C for 15 s; (3) annealing of primers at 60°C for 60 s; steps (2) and (3) were repeated 40 times; and (4) denaturation at 95°C for 15 s, annealing of primers at 60°C for 60 s, and denaturation at 95°C for 15 s. For the mRNA expression analysis, a TaqMan Array 96-Well FAST Plate (Human Stem Cell Pluripotency, Human Maturity-Onset Diabetes, Human Factors Promoting Card; Applied Biosystems) was used. TaqMan Fast Advanced Master Mix (Thermo Fisher Scientific) was used according to the manufacturer’s instructions. The PCR protocol was as follows: (1) denature at 95°C for 20 s; (2) anneal primers at 60°C for 20 s and repeat steps (1) and (2) 40 times.

For the analysis of real-time PCR data using a TaqMan Array96-Well FAST Plate, *18S*, *GAPDH*, *HPRT1*, and *GUSB* were used as housekeeping genes. The maximum CT value was set at 40. The value of ΔCT for undetected targets of CT value was calculated by subtracting the average of the CT values of the four housekeeping genes from the maximum CT value (40). The ΔCT value of the target was calculated by subtracting the average CT values of the four housekeeping genes from the CT values of the various genes under each culture condition. To calculate the ΔΔCT values of the target, the average ΔCT values of the various genes under control culture conditions were subtracted from the ΔCT values of the various genes under each culture condition. The ΔΔCT values were then calculated using an Excel software program (Microsoft Corporation).

Expression was calculated using the ΔΔCt method. The expression of the target gene was normalized against expression of the housekeeping gene. Primers were designed to optimize the sequence for each target, human β-actin, *Brachyury* (*T*), *NKX2*.5, *cardiac muscle* *troponin T* (*cTnT*), *SOX17*, *FOXA2*, *HNF4A*, *PAX6*, *MAP2*, and *SOX1*. The gene names were retrieved from the US National Library of Medicine (National Institutes of Health) website (https://www.ncbi.nlm.nih.gov/pubmed/). The primers for human β-actin, *Brachyury* (*T*), *NKX2*.5, *cardiac muscle* *troponin T* (*cTnT*), *SOX17*, *FOXA2*, *HNF4A*, *PAX6*, *MAP2*, and *SOX1* were designed using the Primer 3 Plus application (http://www.bioinformatics.nl/cgi-bin/primer3plus/primer3plus.cgi). The primers used for PCR have been described previously.^1^^,^^40^^,^^41^^,^^42^ Other primers were purchased from Takara Bio Inc. Oligonucleotide sequences are listed in Table S3.

#### Immunofluorescence staining analyses

Anti-TRA-1-60, Mouse-Mono (TRA-1-60), and NL557, GloLIVE (R&D Systems, Inc.) were used as antibodies to detect TRA-1-60 expressed on the surface of human iPSCs. GloLIVE anti-hTRA-1-60 (NLLC4770R; R&D) was washed twice with culture medium containing the antibody 30 min after the addition of 40 μL of the product solution to 2 mL of medium, according to the manufacturer’s recommended protocol, and then photographed under a fluorescence microscope. Images were recorded using a BZ-X800 fluorescence microscope (Keyence Corporation).

#### PluriTest

Three samples of fourth-passage iPSCs established on atelocollagen beads using SRV iPSC-2 and three samples of fourth-passage iPSCs established on atelocollagen beads using SRV iPSC-4 were used.

The CiRA Foundation (Client) is interested in services provided by the Life Technologies Corporation in the analysis of six (6) client-provided samples using the PluriTest Service. We used the PluriTest analysis service provided by Life Technologies Corporation.^43^ In this assay, 36,000 transcripts and variants against a more than 450-sample reference set are assessed for a gene expression analysis.

The transcriptomes of all samples were analyzed and processed in the PluriTest algorithm to generate pluripotency and novelty scores. The pluripotency score is based on many samples (pluripotent, somatic, and tissues) in the stem cell model matrix, which consists of an extensive reference set of more than 450 cell/tissue types, including 223 hESC (Stem Cell Matrix-2′ database)^24^ lines, 41 iPSC lines, somatic cells, and tissues. Samples with positive pluripotency values are more similar to the pluripotent samples in the model matrix than to other classes of samples in the matrix. The novelty score is based on well-characterized PSCs in the stem cell model matrix. A low novelty score indicates that the tested sample can be well reconstructed based on existing data from other well-characterized iPSC and ESC lines. A high novelty score indicates that there are patterns in the tested sample that cannot be explained by the existing database of well-characterized, karyotypically normal pluripotent stem cells. Partially differentiated pluripotent cells, teratocarcinoma cells, or karyotypically abnormal embryonic stem cells may have a high pluripotency score but cannot be reconstructed well with data from well-characterized, normal pluripotent stem cells and thus are expected to have a high novelty score.

Samples that are flagged as borderline rather than pluripotent are an indication that the tested samples have a molecular signature that is slightly different from the database samples. The database samples are based on a limited number of PSC lines, so it is not entirely surprising that a sample may differ. Since there are many different ways to reprogram cells, core pluripotency may be maintained, but there may be molecular or epigenetic differences affecting a portion of the genes.

RNA purification with this system involves preparing cells with a PureLink RNA Mini Kit (Catalog #12183025; (Thermo Fisher Scientific) and quantification using the NanoDrop2 (Thermo Fisher Scientific). The GeneChip for the PluriTest is prepared using 100 ng total RNA.

#### KaryoStat

Four samples of fourth-passage iPSCs established on atelocollagen beads with SRV iPSC-2 and four samples of fourth-passage iPSCs established on atelocollagen beads with SRV iPSC-4, and as controls, the 15M66 line passaged 17 times on atelocollagen beads and another 15M66 passaged 28 times on iMatrix-511 were used.

The CiRA Foundation (Client) is interested in services provided by the Life Technologies Corporation in the analysis of one client-provided sample using the KaryoStat assay. We used the KaryoStat assay service provided by Life Technologies Corporation.^43^ The KaryoStat assay allows for digital visualization of chromosome aberrations with a resolution similar to g-banding karyotyping. The size of the structural aberration that can be detected is more than 2 Mb for chromosomal gains and more than 1 Mb for chromosomal losses. The KaryoStat array is optimized for balanced whole-genome coverage with a low-resolution DNA CN analysis, and the assay covers all 36,00 0RefSeq genes, including 14,000 OMIM targets. The assay enables the detection of aneuploidies, submicroscopic aberrations, and mosaic events.

Genomic DNA (gDNA) purification cells were prepared using the Genomic DNA Purification Kit (Catalog K 0512 [Qiagen] and quantified using the Qubit dsDNA BR Assay Kit; Catalog Q 32850GeneChip Preparation [Thermo Fisher Scientific]). A total of 250 ng of gDNA was used to prepare the GeneChip for KaryoStat according to the manual, which is an array that looks for CN variants and single-nucleotide polymorphisms across the genome.

gDNA was processed according to the manufacturer’s protocol. In brief, 250 ng gDNA was digested with the restriction enzyme NspI. Digested DNA was then ligated to the Nsp I adapter and amplified via PCR. The PCR products were purified and fragmented with DNase I, and the fragmented products were end-labeled with biotin and hybridized to KaryoStat arrays (Thermo Fisher Scientific) in a GeneChip Hybridization Oven 645 (Thermo Fisher Scientific) overnight. Arrays were washed and stained using a GeneChip Fluidics Station 450 (Thermo Fisher Scientific) and scanned using a GeneChip Scanner 3000 7G (Thermo Fisher Scientific). Scanned data files were generated using the GeneChip Command Console software program and analyzed using the Chromosome Analysis Suite v4.3 (ChAS), considering 1–2 MB for gains/losses and 5 MB for heterozygosity/absence of heterozygosity.

#### Quantification and statistical analyses

Statistical analyses were performed using Student’s *t* test to compare the means of two samples. The analyses of multiple groups (i.e., more than two groups) were performed using one- and two-way analyses of variance with the StatPlus software program (AnalystSoft). Statistical significance was set at ∗*p* < 0.05 or ∗∗*p* < 0.01 for all tests. The data shown are representative examples of two independent experiments.

## Data and code availability

Further information and requests for resources and reagents should be directed to the Lead Contact, Yoshiki Nakashima (yoshiki.nakashima@cira-foundation.or.jp).

Unique materials generated in this study are available from the Lead Contact upon reasonable request following the signing of a Materials Transfer Agreement.

## Supplemental information

Document S1. Figure S1 and Tables S1–S5

Document S2. Article plus supplemental information
