# Supplementary material for: Atelocollagen supports three-dimensional culture of human induced pluripotent stem cells
Source: Mol Ther Methods Clin Dev. 2024 Jul 20;32(3):101302. doi: 10.1016/j.omtm.2024.101302 (PMC11342089; doi:10.1016/j.omtm.2024.101302)
Supplement: Document S1. Figure S1 and Tables S1–S5 [file mmc1.pdf]

OMTM, Volume *32*

Supplemental information

Atelocollagen supports three-dimensional culture of human induced pluripotent stem cells

Yoshiki Nakashima and Masayoshi Tsukahara

Table S1. Gene list in Figure 3G: relative mRNA expression levels

Gene Symbol	Gene name	Control	Pancreatic progenitor	P Value	n
HNF1α	*Hepatocyte nuclear factor 1α*	1.05 ± 0.29	7.81 ± 4.24	< 0.01	6
HNF4α	*Hepatocyte nuclear factor 4α*	1.07 ± 0.38	0.63 ± 0.31	0.06	6
HNF1β	*Hepatocyte nuclear factor 1β*	1.13 ± 0.53	11.20 ± 2.51	< 0.01	6
GCK	Glucokinase	1.20 ± 0.81	3.23 ± 0.92	< 0.01	6
NEUROD1	*Neurogenic differentiation 1*	1.54 ± 1.88	147.16 ± 17.44	< 0.01	6
PDX1	*Pancreas duodenum homeobox 1*	1.14 ± 0.74	0.47 ± 0.11	0.06	6

Relative mRNA expression levels of genes (mean ± standard deviation).

Key factors in pancreatic progenitor cell differentiation maturation were significantly increased in pancreatic progenitor cells induced to differentiate after eight passages of iPSCs in comparison to undifferentiated iPSCs after eight passages.

Table S2. Gene list in Figure 3I: relative mRNA expression levels

Gene Symbol	Gene name	Control	Cardiomyocytes	P Value	n
NKX2.5	NK2 Transcription Factor Related Locus-5	1.01 ± 0.13	31.77 ± 42.49	0.12	6
GATA4	GATA Binding Protein-4	1.00 ± 0.13	76.01 ± 87.89	0.074	6
Tbx5	T-Box-5	1.05 ± 0.40	32.01 ± 26.99	< 0.05	6
Tbx20	T-Box-20	1.05 ± 0.34	137.22 ± 90.07	< 0.01	6
eHAND/HAND1	Heart and Neural crest Derivatives expressed-1	1.01 ± 0.13	904.85 ± 779.81	< 0.05	6
dHAND/HAND2	Heart and Neural crest Derivatives expressed-2	1.00 ± 0.12	60.32 ± 47.23	< 0.05	6
MEF2C	Myocyte Enhancer Factor 2C	1.05 ± 0.39	0.67 ± 0.48	0.18	6
BMP2	Bone Morphogenetic Protein 2	1.01 ± 0.13	5.56 ± 3.93	< 0.05	6
BMP4	Bone Morphogenetic Protein 4	1.00 ± 0.13	4.53 ± 4.50	0.098	6
BMP7	Bone Morphogenetic Protein 7	1.01 ± 0.13	3.13 ± 0.90	< 0.01	6
BMP10	Bone Morphogenetic Protein 10	1.21 ± 0.52	75.78 ± 71.57	< 0.05	6
WNT3A	Wingless-related MMTV integration site 3A	1.66 ± 1.11	541.65 ± 408.46	< 0.05	6
WNT8A	Wingless-related MMTV integration site 8A	1.07 ± 0.38	9.58 ± 7.64	< 0.05	6
WNT8B	Wingless-related MMTV integration site 8B	1.01 ± 0.13	856.59 ± 462.45	< 0.01	6
WNT11	Wingless-related MMTV integration site 11	1.03 ± 0.26	29.23 ± 23.11	< 0.05	6

Relative mRNA expression levels of genes (mean ± standard deviation).

Key factors in cardiomyocytes differentiation maturation were significantly increased in cardiomyocytes induced to differentiate after eight passages of iPSCs in comparison to undifferentiated iPSCs after eight passages.

Table S3. Oligonucleotide sequences list

REAGENT or RESOURCE	SOURCE	IDENTIFIER
Oligonucleotides		
human HNF4a forward, GAACAGGAGCTCTTAACTACAGTGG, human HNF4a reverse, CTGTCAAGAGTCATGAATTCTCCTT,	NCBI Reference Sequence	NM_000457.4
human brachyury (T) forward, GCTGAACTCCTTGCATAAGTATGAG, human brachyury (T) reverse, CATCTCTTTGTGATCACTTCTTTCC,	Nakashima et al. Mol Ther. 2018 Jul 5; 26(7): 1715–1734.	NM_001270484.1
human NKX2.5 forward, GAAATTTTAAGTCACCGTCTGTCTC, human NKX2.5 reverse, AGTAATGGTAAGGGATCCTCGTG,	Nakashima et al. Mol Ther. 2018 Jul 5; 26(7): 1715–1734.	NM_001166175.1
human Troponin T (cTnT) forward, ATGAGCGGGAGAAGGAGCGGCAGAAC, human Troponin T (cTnT) reverse, TCAATGGCCAGCACCTTCCTCCTCTC,	Takahashi, et al. Cell 2007;131:861–872.	NM_001001432.1
human SOX17 forward, CGCTTTCATGGTGTGGGCTAAGGACG, human SOX17 reverse, TAGTTGGGGTGGTCCTGCATGTGCTG,	Takahashi, et al. Cell 2007;131:861–872.	NM_022454.3
human FOXA2 forward, TGGGAGCGGTGAAGATGGAAGGGCAC, human FOXA2 reverse, TCATGCCAGCGCCCACGTACGACGAC,	Takahashi, et al. Cell 2007;131:861–872.	NM_153675.2
human PAX6 forward, ACCCATTATCCAGATGTGTTTGCCCGAG, human PAX6 reverse, ATGGTGAAGCTGGGCATAGGCGGCAG,	Takahashi, et al. Cell 2007;131:861–872.	NM_001604.4
human MAP2 forward, CAGGTGGCGGACGTGTGAAAATTGAGAGTG, human MAP2 reverse, CACGCTGGATCTGCCTGGGGACTGTG,	Takahashi, et al. Cell 2007;131:861–872.	NM_001039538.1
human SOX1 forward, ACTCTCTCTGAGGTTCTTTGACTGA, human SOX1 reverse, AGCTTTTCATAGTCTGTGCCTCTAA,	NCBI Reference Sequence	NM_005986.3
human β-actin forward, TGACATTAAGGAGAAGCTGTGCTAC, human β-actin reverse, CTTCATGATGGAGTTGAAGGTAGTT,	NCBI Reference Sequence	NM_001101.5

Table S4. Gene list in Figure 1D, 1E, 1F, 1G: relative mRNA expression levels

Gene Symbol	Gene name	iMatrix511-coat	iMatrix511	iMatrix511+atero beads	atero beads	iMatrix511+aterocoat	aterocoat	n
DNMT3B	DNA (cytosine-5-)-methyltransferase 3 beta	1.04 ± 0.31	1.28 ± 0.24	0.94 ± 0.16	1.03 ± 0.35	0.79 ± 0.18	0.80 ± 0.26	6
GABRB3	Gamma-aminobutyric acid receptor subunit beta-3	1.02 ± 0.22	1.45 ± 0.28	0.86 ± 0.27	1.00 ± 0.27	0.73 ± 0.22	0.78 ± 0.10	6
GDF3	Growth differentiation factor-3	1.01 ± 0.13	0.90 ± 0.16	0.75 ± 0.22	0.71 ± 0.19	0.76 ± 0.37	0.63 ± 0.16	6
NANOG	Nanog homeobox	1.04 ± 0.30	1.28 ± 0.24	0.59 ± 0.17	0.89 ± 0.24	0.49 ± 0.26	0.75 ± 0.31	6
POU5F1	POU domain, class 5, transcription factor 1	1.04 ± 0.30	1.83 ± 0.99	0.66 ± 0.31	1.52 ± 0.83	0.60 ± 0.62	1.16 ± 0.54	6
SOX2	SRY-box transcription factor 2	1.12 ± 0.61	1.75 ± 0.73	0.92 ± 0.14	1.03 ± 0.33	0.99 ± 0.44	1.44 ± 0.44	6
TDGF1	Teratocarcinoma-derived growth factor 1	1.01 ± 0.13	1.18 ± 0.42	0.75 ± 0.21	0.91 ± 0.28	0.56 ± 0.16	0.43 ± 0.06	6
AFP	Alpha-fetoprotein	1.13 ± 0.59	1.09 ± 0.45	0.66 ± 0.18	0.19 ± 0.18	1.00 ± 0.52	0.38 ± 0.27	6
CTNNB1	Catenin Beta 1	1.02 ± 0.23	1.03 ± 0.26	1.04 ± 0.18	1.00 ± 0.27	0.78 ± 0.14	0.78 ± 0.10	6
FOXA2	Forkhead Box A2	1.02 ± 0.22	1.45 ± 0.28	0.31 ± 0.11	1.72 ± 0.88	0.71 ± 0.18	1.44 ± 0.39	6
GATA4	GATA Binding Protein 4	1.04 ± 0.31	1.52 ± 0.58	1.05 ± 0.15	2.14 ± 0.79	0.65 ± 0.20	1.26 ± 0.28	6
GATA6	GATA Binding Protein 6	1.04 ± 0.32	1.28 ± 0.24	1.15 ± 0.32	1.75 ± 0.47	1.03 ± 0.44	1.26 ± 0.34	6
GCG	Glucagon	1.53 ± 1.25	2.56 ± 1.63	1.44 ± 1.44	0.90 ± 1.28	0.59 ± 0.56	0.61 ± 0.61	6
MNX1	Motor Neuron And Pancreas Homeobox 1	1.12 ± 0.57	2.04 ± 0.36	0.98 ± 0.36	1.27 ± 0.34	0.59 ± 0.32	0.99 ± 0.13	6
IAPP	Islet Amyloid Polypeptide	1.13 ± 0.55	1.39 ± 0.55	1.06 ± 0.25	0.97 ± 0.46	0.79 ± 0.21	0.70 ± 0.19	6
INS	Insulin	1.04 ± 0.30	1.29 ± 0.24	1.19 ± 0.33	1.00 ± 0.25	0.90 ± 0.25	0.89 ± 0.23	6
PDX1	pancreatic and duodenal homeobox 1	1.36 ± 0.88	1.10 ± 1.20	1.15 ± 0.85	0.25 ± 0.06	0.49 ± 0.63	0.22 ± 0.06	6
KIT	KIT Proto-Oncogene, Receptor Tyrosine Kinase	1.04 ± 0.31	1.29 ± 0.24	0.84 ± 0.20	0.72 ± 0.22	0.57 ± 0.15	0.70 ± 0.09	6
LEFTY1	Left-Right Determination Factor 1	1.05 ± 0.33	1.49 ± 0.43	0.43 ± 0.16	0.64 ± 0.40	0.31 ± 0.16	0.40 ± 0.15	6
NEUROD1	Neuronal Differentiation 1	1.03 ± 0.28	1.28 ± 0.25	0.90 ± 0.34	0.98 ± 0.25	0.73 ± 0.36	0.55 ± 0.30	6
PAX4	Paired Box 4	1.02 ± 0.23	1.41 ± 0.26	0.77 ± 0.28	0.80 ± 0.26	0.66 ± 0.23	0.53 ± 0.25	6
PTF1A	Pancreas transcription factor 1 subunit alpha	1.05 ± 0.35	1.22 ± 0.73	0.90 ± 0.46	0.74 ± 0.42	0.42 ± 0.54	0.65 ± 0.26	6
SERPINA1	Serpin Family A Member 1	1.04 ± 0.31	1.18 ± 0.35	0.77 ± 0.24	0.74 ± 0.23	0.80 ± 0.18	0.80 ± 0.25	6
SOX17	SRY-Box Transcription Factor 17	1.18 ± 0.70	1.56 ± 0.63	0.50 ± 0.33	2.07 ± 1.25	0.63 ± 0.94	1.40 ± 0.77	6
TAT	Tyrosine Aminotransferase	1.04 ± 0.34	1.00 ± 0.67	0.32 ± 0.41	1.00 ± 0.26	0.74 ± 0.35	0.70 ± 0.27	6
ACTC1	Actin Alpha Cardiac Muscle 1	1.05 ± 0.34	1.19 ± 0.43	0.75 ± 0.21	0.63 ± 0.16	0.50 ± 0.12	0.44 ± 0.06	6
CD34	cluster of differentiation 34	1.04 ± 0.31	1.15 ± 0.28	0.86 ± 0.24	1.09 ± 0.47	0.79 ± 0.18	0.69 ± 0.09	6
CDH5	Cadherin 5	1.02 ± 0.22	1.46 ± 0.27	1.05 ± 0.18	0.82 ± 0.25	0.79 ± 0.14	0.65 ± 0.21	6
COL1A1	Collagen Type I Alpha 1 Chain	1.04 ± 0.30	1.29 ± 0.25	0.53 ± 0.07	0.42 ± 0.16	0.46 ± 0.18	0.40 ± 0.13	6
COL2A1	Collagen Type II Alpha 1 Chain	1.04 ± 0.31	1.28 ± 0.24	0.52 ± 0.07	0.54 ± 0.23	0.40 ± 0.09	0.40 ± 0.12	6
DDX4	DEAD-Box Helicase 4	1.04 ± 0.31	1.29 ± 0.24	0.92 ± 0.49	0.58 ± 0.19	0.60 ± 0.29	0.45 ± 0.12	6
DES	Desmin	1.04 ± 0.31	1.29 ± 0.25	1.21 ± 0.35	2.34 ± 0.75	1.60 ± 0.37	1.62 ± 0.51	6
FLT1	Fms Related Receptor Tyrosine Kinase 1	1.02 ± 0.22	1.60 ± 0.28	0.96 ± 0.28	0.93 ± 0.35	0.70 ± 0.12	0.89 ± 0.28	6
FN1	Fibronectin 1	1.04 ± 0.30	0.91 ± 0.23	0.95 ± 0.24	0.83 ± 0.31	0.79 ± 0.18	0.69 ± 0.09	6
HBZ	Hemoglobin Subunit Zeta	1.04 ± 0.30	1.54 ± 0.75	0.57 ± 0.36	1.60 ± 0.79	0.55 ± 0.63	1.14 ± 0.52	6
ISL1	ISL LIM Homeobox 1	1.07 ± 0.37	0.92 ± 0.27	0.48 ± 0.15	0.66 ± 0.24	0.40 ± 0.20	0.20 ± 0.06	6
MYF5	Myogenic factor 5	1.03 ± 0.28	2.06 ± 0.46	1.01 ± 0.52	1.13 ± 0.29	0.80 ± 0.40	0.89 ± 0.23	6
MYOD1	Myogenic Differentiation 1	1.58 ± 1.14	1.86 ± 1.17	0.67 ± 0.89	1.25 ± 0.72	1.40 ± 0.97	1.51 ± 0.76	6
PECAM1	Platelet And Endothelial Cell Adhesion Molecule 1	1.02 ± 0.22	1.32 ± 0.38	0.58 ± 0.08	1.00 ± 0.27	0.47 ± 0.18	0.64 ± 0.30	6
RAF1	Raf-1 Proto-Oncogene, Serine/Threonine Kinase	1.04 ± 0.31	1.28 ± 0.24	0.94 ± 0.16	1.34 ± 0.55	0.70 ± 0.12	1.07 ± 0.44	6
RUNX2	RUNX Family Transcription Factor 2	1.04 ± 0.31	1.30 ± 0.24	1.04 ± 0.15	0.69 ± 0.35	0.74 ± 0.26	0.40 ± 0.13	6
SYCP3	Synaptonemal Complex Protein 3	1.13 ± 0.65	2.77 ± 1.13	1.22 ± 0.38	1.15 ± 0.35	1.18 ± 0.40	1.27 ± 0.40	6
T	T-box transcription factor T	1.05 ± 0.33	1.90 ± 0.74	0.59 ± 0.09	4.84 ± 2.16	0.73 ± 0.26	1.60 ± 0.43	6
WT1	Wilms tumor 1	1.02 ± 0.22	1.45 ± 0.27	0.79 ± 0.37	0.81 ± 0.25	0.56 ± 0.15	0.72 ± 0.20	6
BXDC2	brix domain-containing protein 2	1.04 ± 0.30	1.29 ± 0.25	1.04 ± 0.15	1.01 ± 0.34	0.64 ± 0.19	0.80 ± 0.25	6
CD9	CD9 Molecule	1.04 ± 0.31	0.81 ± 0.17	1.20 ± 0.35	1.27 ± 0.29	1.14 ± 0.31	0.90 ± 0.24	6
CGB	Chorionic Gonadotropin Subunit Beta 3	1.14 ± 0.59	1.02 ± 0.19	0.47 ± 0.13	0.39 ± 0.20	0.58 ± 0.20	0.47 ± 0.18	6
COMMD3,BMI1	COMMD3-BMI1 Readthrough	1.04 ± 0.30	1.48 ± 0.46	0.83 ± 0.14	0.80 ± 0.21	0.63 ± 0.18	1.16 ± 0.34	6
CRABP2	Cellular Retinoic Acid Binding Protein 2	1.02 ± 0.22	1.32 ± 0.39	0.86 ± 0.27	1.01 ± 0.27	0.89 ± 0.20	0.90 ± 0.28	6
LEFTY2	Left-Right Determination Factor 2	1.04 ± 0.30	2.02 ± 0.94	0.69 ± 0.26	1.53 ± 0.84	0.72 ± 0.58	1.16 ± 0.54	6
EEF1A1	Eukaryotic Translation Elongation Factor 1 Alpha 1	1.04 ± 0.31	1.21 ± 0.39	0.61 ± 0.24	0.77 ± 0.37	0.44 ± 0.25	0.76 ± 0.31	6
FGF4	Fibroblast Growth Factor 4	1.04 ± 0.31	1.47 ± 0.41	1.01 ± 0.46	1.56 ± 0.73	0.80 ± 0.51	1.30 ± 0.37	6
FGF5	Fibroblast Growth Factor 5	1.04 ± 0.30	1.49 ± 0.57	0.80 ± 0.34	0.66 ± 0.25	0.62 ± 0.10	1.01 ± 0.72	6
FOXD3	Forkhead Box D3	1.08 ± 0.43	1.45 ± 0.28	0.59 ± 0.29	1.65 ± 0.96	0.62 ± 0.71	1.29 ± 0.59	6
GAL	Galanin And GMAP Prepropeptide	1.06 ± 0.38	1.15 ± 0.22	0.76 ± 0.22	1.40 ± 0.66	0.58 ± 0.18	0.43 ± 0.20	6
GBX2	Gastrulation Brain Homeobox 2	1.03 ± 0.23	1.88 ± 0.56	0.48 ± 0.13	0.87 ± 0.37	0.43 ± 0.20	0.60 ± 0.25	6
GFAP	Glial Fibrillary Acidic Protein	1.04 ± 0.30	1.47 ± 0.41	0.97 ± 0.28	1.02 ± 0.32	0.79 ± 0.18	0.81 ± 0.25	6
GRB7	Growth Factor Receptor Bound Protein 7	1.04 ± 0.31	1.50 ± 0.58	1.04 ± 0.15	1.66 ± 0.62	1.48 ± 0.52	2.62 ± 0.75	6
IFITM1	Interferon Induced Transmembrane Protein 1	1.02 ± 0.22	1.45 ± 0.27	1.17 ± 0.16	1.15 ± 0.38	0.89 ± 0.20	0.73 ± 0.21	6
IFITM2	Interferon Induced Transmembrane Protein 2	1.04 ± 0.30	1.28 ± 0.24	0.69 ± 0.25	0.89 ± 0.24	0.47 ± 0.23	0.53 ± 0.22	6
IL6ST	Interleukin 6 Cytokine Family Signal Transducer	1.02 ± 0.22	1.45 ± 0.27	0.77 ± 0.28	1.00 ± 0.27	0.65 ± 0.23	0.78 ± 0.10	6
IGF2BP2	Insulin-like growth factor 2 mRNA-binding protein 2	1.04 ± 0.31	1.88 ± 0.65	1.04 ± 0.15	1.45 ± 0.44	0.70 ± 0.13	1.30 ± 0.35	6
LAMA1	Laminin Subunit Alpha 1	1.04 ± 0.31	1.29 ± 0.24	0.60 ± 0.17	0.66 ± 0.27	0.51 ± 0.17	0.65 ± 0.19	6
LAMB1	Laminin Subunit Beta 1	1.02 ± 0.21	1.14 ± 0.23	0.84 ± 0.24	1.17 ± 0.44	0.88 ± 0.15	0.98 ± 0.13	6
LAMC1	Laminin Subunit Gamma 1	1.02 ± 0.22	0.90 ± 0.18	0.67 ± 0.19	0.93 ± 0.35	0.56 ± 0.15	0.73 ± 0.21	6
LIFR	LIF Receptor Subunit Alpha	1.60 ± 1.16	1.79 ± 1.15	1.18 ± 0.95	1.05 ± 0.89	0.93 ± 0.78	1.52 ± 0.77	6
LIN28	Lin-28 homolog A	1.05 ± 0.31	1.68 ± 0.53	0.95 ± 0.27	1.43 ± 0.38	0.71 ± 0.20	1.04 ± 0.30	6
NES	Nestin	1.05 ± 0.34	1.62 ± 0.30	0.61 ± 0.17	0.97 ± 0.46	0.66 ± 0.28	1.34 ± 0.55	6
NODAL	Nodal Growth Differentiation Factor	1.04 ± 0.31	1.47 ± 0.41	0.94 ± 0.16	0.72 ± 0.22	0.79 ± 0.18	0.59 ± 0.20	6
NOG	Noggin	1.12 ± 0.61	1.78 ± 0.83	0.55 ± 0.30	1.43 ± 0.71	0.52 ± 0.56	1.22 ± 0.70	6
NR5A2	nuclear receptor subfamily 5, group A, member 2	1.04 ± 0.31	1.28 ± 0.24	1.20 ± 0.34	1.80 ± 0.48	0.79 ± 0.18	0.69 ± 0.09	6
NR6A1	Nuclear Receptor Subfamily 6 Group A Member 1	1.04 ± 0.31	1.47 ± 0.40	0.85 ± 0.20	1.16 ± 0.37	0.64 ± 0.19	0.80 ± 0.25	6
OLIG2	Oligodendrocyte Transcription Factor 2	1.05 ± 0.31	1.66 ± 0.51	1.08 ± 0.39	1.14 ± 0.36	1.05 ± 0.48	1.10 ± 0.14	6
PAX6	Paired Box 6	1.06 ± 0.38	2.29 ± 0.43	1.07 ± 0.30	1.38 ± 0.66	0.71 ± 0.16	1.24 ± 0.16	6
PODXL	Podocalyxin Like	1.02 ± 0.22	1.32 ± 0.39	0.67 ± 0.19	0.93 ± 0.35	0.50 ± 0.14	0.73 ± 0.21	6
PTEN	Phosphatase And Tensin Homolog	1.04 ± 0.30	2.03 ± 0.99	0.44 ± 0.31	1.47 ± 0.86	0.55 ± 0.64	1.15 ± 0.53	6
REST	RE1 Silencing Transcription Factor	1.05 ± 0.33	1.62 ± 0.31	0.43 ± 0.16	0.84 ± 0.34	0.33 ± 0.14	0.65 ± 0.35	6
SEMA3A	Semaphorin 3A	1.03 ± 0.22	0.90 ± 0.17	0.97 ± 0.37	0.89 ± 0.21	0.55 ± 0.13	0.49 ± 0.06	6
SFRP2	Secreted Frizzled Related Protein 2	1.02 ± 0.22	1.45 ± 0.28	1.05 ± 0.18	1.13 ± 0.29	0.89 ± 0.20	0.78 ± 0.10	6
SYP	Synaptophysin	1.04 ± 0.31	1.31 ± 0.25	1.05 ± 0.15	1.14 ± 0.31	1.03 ± 0.35	0.90 ± 0.24	6
TERT	Telomerase Reverse Transcriptase	1.02 ± 0.22	1.45 ± 0.27	0.76 ± 0.22	0.81 ± 0.25	0.89 ± 0.20	0.78 ± 0.10	6
TFCP2L1	Transcription Factor CP2 Like 1	1.02 ± 0.22	1.44 ± 0.27	0.77 ± 0.28	0.87 ± 0.41	0.72 ± 0.22	0.73 ± 0.21	6
TH	Tyrosine Hydroxylase	1.81 ± 2.00	1.58 ± 2.25	0.60 ± 0.17	1.39 ± 1.23	0.45 ± 0.12	1.23 ± 1.92	6
UTF1	Undifferentiated Embryonic Cell Transcription Factor 1	1.04 ± 0.30	2.16 ± 0.76	0.17 ± 0.10	1.30 ± 0.97	0.68 ± 0.30	1.20 ± 0.45	6
XIST	X-inactive specific transcript	1.04 ± 0.30	1.19 ± 0.36	2.08 ± 0.30	0.89 ± 0.24	1.01 ± 0.27	0.92 ± 0.30	6
ZFP42	ZFP42 Zinc Finger Protein	1.04 ± 0.34	1.89 ± 0.66	1.22 ± 0.44	1.64 ± 0.50	1.03 ± 0.72	1.03 ± 0.34	6

Relative mRNA expression levels of genes (mean ± standard deviation).

Table S5. Gene list in Figure S1F, S1G, S1H, S1I: relative mRNA expression levels

Gene Symbol	Gene name	Vitronectin-coat	Vitronectin	Vitronectin+atero beads	atero beads	Vitronectin+aterocoat	aterocoat	n
DNMT3B	DNA (cytosine-5-)-methyltransferase 3 beta	1.07 ± 0.42	1.48 ± 0.16	1.03 ± 0.52	0.42 ± 0.10	0.92 ± 0.27	0.93 ± 0.16	6
GABRB3	Gamma-aminobutyric acid receptor subunit beta-3	1.04 ± 0.28	1.33 ± 0.30	1.35 ± 0.25	0.47 ± 0.13	0.63 ± 0.06	0.81 ± 0.23	6
GDF3	Growth differentiation factor-3	1.08 ± 0.42	1.32 ± 0.14	0.89 ± 0.28	0.93 ± 0.19	0.93 ± 0.28	0.95 ± 0.12	6
NANOG	Nanog homeobox	1.04 ± 0.27	1.18 ± 0.64	1.22 ± 0.23	0.54 ± 0.14	0.65 ± 0.19	0.84 ± 0.26	6
POU5F1	POU domain, class 5, transcription factor 1	1.03 ± 0.29	1.88 ± 0.42	1.16 ± 0.56	0.16 ± 0.06	0.83 ± 0.21	0.87 ± 0.46	6
SOX2	SRY-box transcription factor 2	1.00 ± 0.09	1.04 ± 0.24	1.97 ± 0.58	1.12 ± 0.45	1.19 ± 0.50	1.02 ± 0.40	6
TDGF1	Teratocarcinoma-derived growth factor 1	1.03 ± 0.30	1.38 ± 0.45	1.08 ± 0.19	0.67 ± 0.18	0.81 ± 0.17	1.00 ± 0.19	6
AFP	Alpha-fetoprotein	1.06 ± 0.43	1.50 ± 0.37	1.61 ± 1.06	0.45 ± 0.22	0.59 ± 0.23	0.78 ± 1.23	6
CTNNB1	Catenin Beta 1	1.06 ± 0.34	1.16 ± 0.13	1.11 ± 0.35	0.83 ± 0.16	0.72 ± 0.21	0.81 ± 0.23	6
FOXA2	Forkhead Box A2	1.04 ± 0.27	1.23 ± 0.42	0.56 ± 0.17	0.77 ± 0.24	1.26 ± 0.12	1.20 ± 0.48	6
GATA4	GATA Binding Protein 4	1.08 ± 0.42	1.21 ± 0.38	1.19 ± 0.52	0.85 ± 0.43	1.35 ± 0.52	0.83 ± 0.38	6
GATA6	GATA Binding Protein 6	1.00 ± 0.09	1.51 ± 0.39	1.59 ± 0.56	1.44 ± 1.08	1.56 ± 1.39	1.09 ± 0.25	6
GCG	Glucagon	1.21 ± 0.63	0.88 ± 1.01	1.34 ± 1.14	1.87 ± 1.73	0.82 ± 0.33	1.03 ± 0.48	6
MNX1	Motor Neuron And Pancreas Homeobox 1	1.08 ± 0.41	1.27 ± 0.56	1.44 ± 0.64	0.40 ± 0.34	1.40 ± 0.13	1.25 ± 0.45	6
IAPP	Islet Amyloid Polypeptide	1.03 ± 0.30	1.55 ± 0.43	0.66 ± 0.30	1.08 ± 0.33	1.06 ± 0.43	1.79 ± 0.73	6
INS	Insulin	1.87 ± 1.77	0.57 ± 0.73	1.51 ± 2.33	0.05 ± 0.03	0.01 ± 0.00	0.02 ± 0.00	6
PDX1	pancreatic and duodenal homeobox 1	1.70 ± 1.98	0.65 ± 0.15	3.94 ± 7.88	11.43 ± 15.86	0.31 ± 0.03	5.17 ± 6.07	6
KIT	KIT Proto-Oncogene, Receptor Tyrosine Kinase	1.06 ± 0.41	1.35 ± 0.33	1.25 ± 0.44	0.52 ± 0.10	0.89 ± 0.45	0.70 ± 0.58	6
LEFTY1	Left-Right Determination Factor 1	1.17 ± 0.60	1.66 ± 0.38	0.97 ± 0.57	0.57 ± 0.26	0.80 ± 0.07	0.93 ± 0.53	6
NEUROD1	Neuronal Differentiation 1	1.04 ± 0.25	1.20 ± 0.27	1.69 ± 0.76	1.27 ± 0.87	0.84 ± 0.28	0.84 ± 0.27	6
PAX4	Paired Box 4	1.11 ± 0.52	1.05 ± 0.58	2.98 ± 3.46	0.14 ± 0.25	0.43 ± 0.48	1.07 ± 0.97	6
PTF1A	Pancreas transcription factor 1 subunit alpha	1.07 ± 0.38	2.58 ± 2.87	9.90 ± 6.74	4.13 ± 4.11	4.42 ± 5.70	2.63 ± 3.10	6
SERPINA1	Serpin Family A Member 1	1.00 ± 0.09	1.04 ± 0.25	1.57 ± 0.57	1.74 ± 0.58	2.08 ± 1.24	1.24 ± 0.73	6
SOX17	SRY-Box Transcription Factor 17	1.11 ± 0.54	3.46 ± 1.13	1.06 ± 0.88	0.13 ± 0.09	0.65 ± 0.38	0.76 ± 0.94	6
TAT	Tyrosine Aminotransferase	1.56 ± 1.11	1.04 ± 1.03	0.81 ± 0.60	1.58 ± 1.37	0.77 ± 0.64	0.76 ± 0.74	6
ACTC1	Actin Alpha Cardiac Muscle 1	1.08 ± 0.42	1.30 ± 0.14	2.60 ± 1.16	0.87 ± 0.29	0.71 ± 0.07	0.70 ± 0.69	6
CD34	cluster of differentiation 34	1.05 ± 0.35	1.27 ± 0.55	2.94 ± 3.80	0.08 ± 0.04	0.23 ± 0.49	0.03 ± 0.00	6
CDH5	Cadherin 5	1.00 ± 0.10	1.05 ± 0.24	1.36 ± 0.28	0.66 ± 0.13	0.75 ± 0.25	0.74 ± 0.38	6
COL1A1	Collagen Type I Alpha 1 Chain	1.00 ± 0.09	1.54 ± 0.51	1.09 ± 0.29	0.16 ± 0.03	1.00 ± 0.09	0.68 ± 0.41	6
COL2A1	Collagen Type II Alpha 1 Chain	1.06 ± 0.41	1.34 ± 0.33	0.77 ± 0.18	0.24 ± 0.07	0.79 ± 0.07	0.76 ± 0.56	6
DDX4	DEAD-Box Helicase 4	1.07 ± 0.37	0.96 ± 0.66	1.25 ± 0.59	1.16 ± 1.08	0.84 ± 0.32	0.81 ± 0.24	6
DES	Desmin	1.03 ± 0.30	1.65 ± 0.19	1.21 ± 0.25	0.67 ± 0.18	0.73 ± 0.20	1.06 ± 0.41	6
FLT1	Fms Related Receptor Tyrosine Kinase 1	1.03 ± 0.22	0.95 ± 0.24	1.96 ± 0.60	0.47 ± 0.16	0.64 ± 0.19	0.76 ± 0.31	6
FN1	Fibronectin 1	1.04 ± 0.26	1.17 ± 0.27	1.54 ± 0.31	0.75 ± 0.15	0.83 ± 0.22	0.77 ± 0.30	6
HBZ	Hemoglobin Subunit Zeta	1.08 ± 0.43	2.10 ± 0.47	1.02 ± 0.76	0.28 ± 0.17	0.94 ± 0.24	0.91 ± 0.38	6
ISL1	ISL LIM Homeobox 1	1.08 ± 0.42	1.09 ± 0.36	0.78 ± 0.23	0.38 ± 0.14	0.35 ± 0.03	0.79 ± 0.26	6
MYF5	Myogenic factor 5	1.68 ± 1.53	1.92 ± 1.47	3.33 ± 2.01	6.16 ± 9.93	3.94 ± 1.00	2.80 ± 1.94	6
MYOD1	Myogenic Differentiation 1	1.13 ± 0.57	1.08 ± 0.55	1.51 ± 1.14	0.63 ± 0.52	1.11 ± 0.11	1.06 ± 0.33	6
PECAM1	Platelet And Endothelial Cell Adhesion Molecule 1	1.04 ± 0.27	1.27 ± 0.74	1.41 ± 0.42	0.24 ± 0.08	0.63 ± 0.06	0.94 ± 0.73	6
RAF1	Raf-1 Proto-Oncogene, Serine/Threonine Kinase	1.06 ± 0.34	1.16 ± 0.13	1.51 ± 0.26	0.42 ± 0.13	1.16 ± 0.29	0.81 ± 0.24	6
RUNX2	RUNX Family Transcription Factor 2	1.07 ± 0.42	1.57 ± 0.55	2.17 ± 0.44	2.08 ± 0.43	1.77 ± 0.89	1.03 ± 0.12	6
SYCP3	Synaptonemal Complex Protein 3	1.02 ± 0.21	1.17 ± 0.27	2.32 ± 1.04	4.21 ± 1.12	2.31 ± 1.35	1.65 ± 0.85	6
T	T-box transcription factor T	1.06 ± 0.33	1.24 ± 0.42	1.43 ± 0.59	1.00 ± 0.76	0.91 ± 0.64	0.92 ± 0.31	6
WT1	Wilms tumor 1	1.06 ± 0.42	1.36 ± 0.33	1.26 ± 0.44	1.36 ± 0.46	1.46 ± 0.30	1.13 ± 0.53	6
BXDC2	brix domain-containing protein 2	1.00 ± 0.09	1.70 ± 0.43	1.36 ± 0.28	0.95 ± 0.26	0.75 ± 0.26	0.91 ± 0.39	6
CD9	CD9 Molecule	1.03 ± 0.22	1.04 ± 0.11	1.09 ± 0.27	0.84 ± 0.23	0.76 ± 0.30	0.77 ± 0.30	6
CGB	Chorionic Gonadotropin Subunit Beta 3	1.02 ± 0.21	1.89 ± 0.46	2.01 ± 0.81	1.00 ± 0.54	0.82 ± 0.22	0.87 ± 0.34	6
COMMD3,BMI1	COMMD3-BMI1 Readthrough	1.00 ± 0.09	1.05 ± 0.24	1.62 ± 0.74	2.20 ± 0.85	1.65 ± 0.44	1.22 ± 0.25	6
CRABP2	Cellular Retinoic Acid Binding Protein 2	1.00 ± 0.09	1.05 ± 0.24	0.97 ± 0.23	0.66 ± 0.13	0.67 ± 0.26	0.74 ± 0.38	6
LEFTY2	Left-Right Determination Factor 2	1.07 ± 0.38	1.93 ± 0.64	1.26 ± 0.96	0.70 ± 0.27	0.79 ± 0.38	0.75 ± 0.29	6
EEF1A1	Eukaryotic Translation Elongation Factor 1 Alpha 1	1.00 ± 0.09	1.95 ± 0.67	2.15 ± 0.40	0.75 ± 0.20	0.83 ± 0.26	0.91 ± 0.39	6
FGF4	Fibroblast Growth Factor 4	1.04 ± 0.27	1.18 ± 0.26	1.25 ± 0.38	1.03 ± 0.56	0.83 ± 0.28	1.09 ± 0.38	6
FGF5	Fibroblast Growth Factor 5	1.06 ± 0.43	1.73 ± 0.60	2.04 ± 0.81	1.88 ± 1.54	1.62 ± 0.35	1.89 ± 0.70	6
FOXD3	Forkhead Box D3	1.03 ± 0.29	1.87 ± 0.42	1.15 ± 0.56	0.12 ± 0.08	0.74 ± 0.23	0.87 ± 0.46	6
GAL	Galanin And GMAP Prepropeptide	1.07 ± 0.38	1.32 ± 0.30	0.99 ± 0.34	0.96 ± 0.35	0.63 ± 0.06	0.81 ± 0.23	6
GBX2	Gastrulation Brain Homeobox 2	1.03 ± 0.22	1.18 ± 0.27	0.77 ± 0.44	0.18 ± 0.04	0.75 ± 0.29	0.77 ± 0.31	6
GFAP	Glial Fibrillary Acidic Protein	1.03 ± 0.30	1.33 ± 0.80	1.12 ± 0.35	0.54 ± 0.17	0.74 ± 0.19	0.80 ± 0.48	6
GRB7	Growth Factor Receptor Bound Protein 7	1.05 ± 0.33	1.15 ± 0.12	1.22 ± 0.29	0.47 ± 0.13	0.73 ± 0.21	0.84 ± 0.78	6
IFITM1	Interferon Induced Transmembrane Protein 1	1.03 ± 0.29	0.93 ± 0.21	1.21 ± 0.24	0.90 ± 0.43	0.51 ± 0.15	0.71 ± 0.48	6
IFITM2	Interferon Induced Transmembrane Protein 2	1.04 ± 0.27	1.35 ± 0.43	1.77 ± 0.62	1.16 ± 0.58	0.67 ± 0.27	0.82 ± 0.90	6
IL6ST	Interleukin 6 Cytokine Family Signal Transducer	1.03 ± 0.30	1.66 ± 0.18	1.21 ± 0.25	0.60 ± 0.19	0.90 ± 0.09	1.01 ± 0.19	6
IGF2BP2	Insulin-like growth factor 2 mRNA-binding protein 2	1.07 ± 0.38	1.14 ± 0.50	1.22 ± 0.28	0.30 ± 0.10	0.69 ± 0.34	0.81 ± 0.23	6
LAMA1	Laminin Subunit Alpha 1	1.04 ± 0.27	1.16 ± 0.13	1.00 ± 0.35	0.94 ± 0.25	1.28 ± 0.14	1.21 ± 0.50	6
LAMB1	Laminin Subunit Beta 1	1.03 ± 0.22	1.03 ± 0.11	1.09 ± 0.26	0.65 ± 0.09	0.84 ± 0.29	0.84 ± 0.26	6
LAMC1	Laminin Subunit Gamma 1	1.00 ± 0.09	0.92 ± 0.10	0.97 ± 0.23	0.37 ± 0.10	0.82 ± 0.22	0.91 ± 0.40	6
LIFR	LIF Receptor Subunit Alpha	1.45 ± 1.01	1.51 ± 0.89	1.20 ± 1.18	0.49 ± 0.27	0.29 ± 0.35	1.26 ± 2.12	6
LIN28	Lin-28 homolog A	1.00 ± 0.09	1.05 ± 0.24	1.08 ± 0.20	0.19 ± 0.05	0.93 ± 0.24	0.85 ± 0.44	6
NES	Nestin	1.03 ± 0.30	1.89 ± 0.43	1.08 ± 0.19	0.34 ± 0.09	1.03 ± 0.30	1.01 ± 0.20	6
NODAL	Nodal Growth Differentiation Factor	1.04 ± 0.26	1.38 ± 0.57	1.24 ± 0.38	1.22 ± 0.48	1.19 ± 0.46	0.96 ± 0.36	6
NOG	Noggin	1.06 ± 0.41	1.66 ± 0.37	1.02 ± 0.49	0.13 ± 0.06	0.66 ± 0.21	0.84 ± 0.55	6
NR5A2	nuclear receptor subfamily 5, group A, member 2	1.04 ± 0.27	1.17 ± 0.27	1.36 ± 0.24	0.74 ± 0.15	0.84 ± 0.28	1.20 ± 0.33	6
NR6A1	Nuclear Receptor Subfamily 6 Group A Member 1	1.03 ± 0.22	1.18 ± 0.28	1.08 ± 0.26	0.27 ± 0.09	0.56 ± 0.05	0.77 ± 0.30	6
OLIG2	Oligodendrocyte Transcription Factor 2	1.06 ± 0.43	0.94 ± 0.21	0.71 ± 0.25	0.68 ± 0.25	1.06 ± 0.43	0.77 ± 0.44	6
PAX6	Paired Box 6	1.16 ± 0.68	1.18 ± 0.27	1.65 ± 0.74	1.84 ± 1.10	1.46 ± 1.61	0.77 ± 0.30	6
PODXL	Podocalyxin Like	1.03 ± 0.30	1.24 ± 0.48	1.41 ± 0.50	0.59 ± 0.12	0.82 ± 0.21	0.87 ± 0.46	6
PTEN	Phosphatase And Tensin Homolog	1.06 ± 0.41	2.08 ± 1.00	1.33 ± 1.40	0.16 ± 0.14	2.01 ± 0.97	1.64 ± 1.28	6
REST	RE1 Silencing Transcription Factor	1.06 ± 0.41	1.47 ± 0.17	1.13 ± 0.47	0.59 ± 0.16	1.60 ± 0.16	1.44 ± 0.61	6
SEMA3A	Semaphorin 3A	1.00 ± 0.09	0.91 ± 0.10	1.37 ± 0.27	0.66 ± 0.13	1.08 ± 0.42	0.73 ± 0.39	6
SFRP2	Secreted Frizzled Related Protein 2	1.08 ± 0.42	1.20 ± 0.30	0.96 ± 0.19	0.94 ± 0.19	1.08 ± 0.42	0.86 ± 0.18	6
SYP	Synaptophysin	1.03 ± 0.22	1.18 ± 0.27	1.41 ± 0.45	1.27 ± 0.52	1.03 ± 0.21	0.98 ± 0.39	6
TERT	Telomerase Reverse Transcriptase	1.00 ± 0.10	1.05 ± 0.24	1.23 ± 0.34	0.75 ± 0.20	0.91 ± 0.19	1.09 ± 0.25	6
TFCP2L1	Transcription Factor CP2 Like 1	1.06 ± 0.33	1.16 ± 0.13	1.07 ± 0.52	0.44 ± 0.17	1.31 ± 0.78	1.01 ± 0.23	6
TH	Tyrosine Hydroxylase	1.04 ± 0.27	1.15 ± 0.26	1.31 ± 0.55	0.67 ± 0.58	1.01 ± 0.21	1.08 ± 0.36	6
UTF1	Undifferentiated Embryonic Cell Transcription Factor 1	1.28 ± 0.94	1.63 ± 1.32	0.74 ± 0.50	0.82 ± 0.11	3.57 ± 2.13	5.16 ± 5.11	6
XIST	X-inactive specific transcript	1.04 ± 0.26	1.10 ± 0.38	1.52 ± 0.32	1.14 ± 0.54	1.01 ± 0.76	0.84 ± 0.26	6
ZFP42	ZFP42 Zinc Finger Protein	1.27 ± 0.90	2.25 ± 1.51	2.17 ± 0.51	1.38 ± 0.88	2.24 ± 0.21	0.94 ± 0.26	6

Relative mRNA expression levels of genes (mean ± standard deviation).

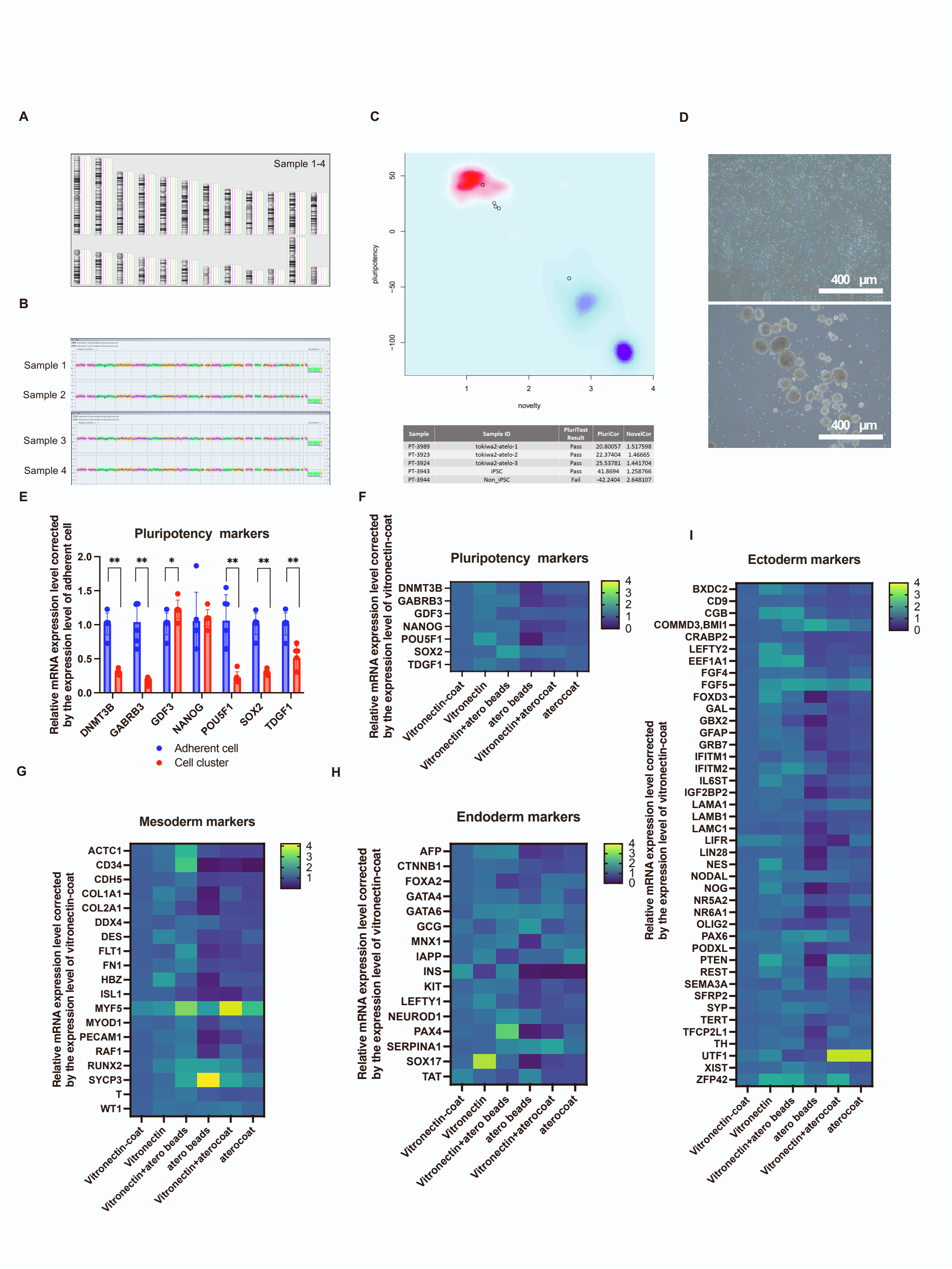

Figure S1. Evaluation of mRNA expression, pluripotency, and karyotype using atelocollagen-based culture methods. Whole-genome view (WGV) of samples: The WGV displays all somatic and sex chromosomes with a high copy number in a single frame. The smooth signal plot (right y-axis) is the smoothing of the log 2 ratios, which depict the signal intensities of probes on the microarray. A value of 2 represents an abnormal copy number state (CN 2), a value of 3 represents chromosomal gain (CN

3), and a value of 1 represents a chromosomal loss (CN 1). The pink, green, and yellow colors indicate the raw signal for each individual chromosome probe, while the blue signal represents the normalized probe signal, which is used to identify copy number and aberrations. Karyoview is a visualization of the WGV reconstructed into chromosome positions (A and B). PluriTest™ results. Three samples of passage 4 iPSCs established on atelocollagen beads using the SRV iPSC-2 vector were used. The samples were analyzed using an algorithm that integrates gene expression data to authenticate pluripotency status. Samples were screened against samples in the stem cell database and given a pluripotency score (PluriCor) and novelty score (NovelCor), which are shown in the table. Pass indicates a clear pluripotency signature, while Fail means that the samples are not pluripotent. A non-iPSC sample was used in this experiment to serve as a negative control for nonpluripotency (C). Optical microscope images 6 days after seeding of PBMC-derived iPSCs at 1.3 × 104 cells/well onto Normal 6-well Plates (upper panel) or Costar® 6-well Clear Flat Bottom Ultra-Low Attachment Multiple Well Plates (lower panel) with the reagent iMatrix-511 at 4.8 µl (0.5 µg/µl)/well. Scale bar = 400 µm (D), and mRNA expression analysis results (n = 6 independent and separate experiments). A real-time qPCR analysis of the pluripotency marker. *P < 0.05. **P < 0.01 (E). mRNA expression analysis results from PBMC-derived iPSCs at day 6 after cell seeding at 1.3 × 104 cells/well. Cultured iPSCs were seeded (1) on vitronectin-coated plates, (2) in vitronectin-containing medium, (3) in vitronectin-containing medium with atelocollagen beads, (4) with atelocollagen beads, (5) in vitronectin-containing medium on atelocollagen-coated plates, (6) on atelocollagen-coated plates. The cDNAs were synthesized from iPSCs sampled 6 days after seeding. Expression levels were calculated using the ΔΔCt method. The expression of the target gene was normalized against the expression of the housekeeping gene. Data were normalized by converting the average expression of various mRNAs of iPSCs cultured on vitronectin-coat to 1. A real-time qPCR analysis of the pluripotency marker (F), expression of mesoderm marker (G), endoderm markers (H) and ectoderm marker (I) is shown (n = 6 independent and separate experiments).
